# The effects of blast-induced traumatic brain injury on brain cellular mechanics and differentiation

**DOI:** 10.1371/journal.pone.0355739

**Published:** 2026-08-13

**Authors:** Nabila Masud, Catherine Fonder, Bridget McGovern, Md Hasibul Hasan Hasib, William J. Jackson, Dulce C. Resendiz, Carley Rivers, Sarah A. Bentil, Donald S. Sakaguchi, Anwesha Sarkar

**Affiliations:** 1 Electrical and Computer Engineering, Iowa State University, Ames, Iowa, United States of America; 2 Molecular, Cellular, and Developmental Biology Program, Iowa State University, Ames, Iowa, United States of America; 3 Department of Genetics, Development, and Cell Biology, Iowa State University, Ames, Iowa, United States of America; 4 Nanovaccine Institute, Iowa State University, Ames, Iowa, United States of America; 5 Biology Program, Iowa State University, Ames, Iowa, United States of America; 6 Mechanical Engineering, Iowa State University, Ames, Iowa, United States of America; 7 Neuroscience Graduate Program, Iowa State University, Ames, Iowa, United States of America; Lady Hardinge Medical College, INDIA

## Abstract

Blast-induced traumatic brain injury (bTBI) causes significant disruptions in cellular and subcellular structures within the central nervous system (CNS) when an extremely large force is applied. The corresponding changes in biomechanical properties and cellular functionalities of neuronal and glial cells due to bTBI remain largely unexplored. In this work, high blast overpressures (BOPs) of 14.5 psi (single shockwave) and 29.0 psi (double shockwave) were applied to adult hippocampal progenitor cells (AHPCs) in two different directions (overpressure applied from ‘top-to-bottom’ and ‘bottom-to-top’ direction on the cell culture petridish). The resultant alterations in structural, nanomechanical, and viscoelastic properties as well as cellular survival, proliferation, and differentiation were analyzed using atomic force microscopy (AFM) and immunocytochemistry (ICC). Double shockwave exposure from ‘bottom-to-top’ direction yielded reduced Young’s modulus, surface roughness, and viscosity, causing significant actin cytoskeletal disruptions compared to ‘top-to-bottom’ direction. ICC results demonstrated that double shockwave exposure from ‘top-to-bottom’ direction caused populations of oligodendrocytes and immature neurons to decrease, while ‘bottom-to-top’ double shockwave exposure caused an increase in the percentage of immature neurons as shown by increased TuJ1-immunoreactivity which is interpreted as evidence that cells have committed to a neuronal lineage and entered an immature/early neuronal stage. These findings emphasize the interplay among cellular differentiation, mechanics, and resilience of neuronal and glial cells to trauma in bTBI aftermath.

## Introduction

**Fig 1:**
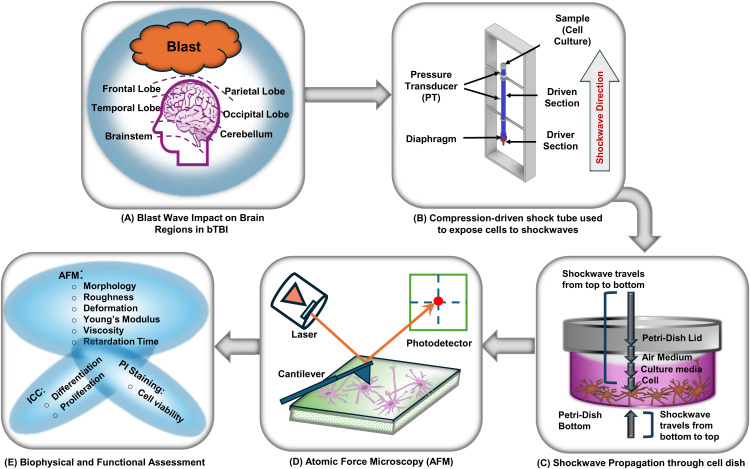
Schematic workflow for in vitro modeling and assessment of blast-induced traumatic brain injury (bTBI). (A) Illustration of a blast wave injury to different regions of the brain, i.e., frontal, parietal, temporal, and occipital lobes, and cerebellum and brainstem, showing the widespread nature of the effect of bTBI. (B) Schematic of a shock tube device employed to produce controlled shockwaves. It has a driven section (blue) holding cell cultures (light blue) and a driver section (red), divided by a diaphragm. When activated by pressurizing the driver section with compressed air, the diaphragm bursts and creates the shockwave that propagates through the driven section towards the cells. The cells within the substrates are clamped to the end of the driven section of the shock tube to facilitate shockwave exposure. (C) Schematic showing the transmission of the shockwave through a Petri dish of culture-grown cells. The shockwave is transmitted from top-to-bottom and bottom-to-top, interacting with cells. (D) Illustration of AFM where a cell surface is probed by a cantilever and deflection is measured by a laser-photodetector system to calculate nanoscale alterations. (E) List of parameters assessed by AFM, including cell morphology, surface roughness, deformation, Young’s modulus, viscosity, and retardation time. Functional impacts such as cell viability, differentiation, and proliferation were also measured by PI staining and ICC study to assess the cellular response to bTBI.

According to the Glasgow Coma Scale (GCS), traumatic brain injury (TBI) is commonly classified by clinical severity, with mild (13–15), moderate (9–12), or severe (≤8) [1,2]. These mild TBIs (mTBIs) can cause a variety of symptoms, such as difficulty focusing, blurred vision, irritability, headaches, sleep problems, and depression, and are difficult to diagnose [3]. In particular, bTBI is a major cause of death and disability in the United States and is often caused by explosions in war zones [1,2,4]. Unlike blunt force trauma and neurodegenerative diseases, this bTBI occurs when extreme mechanical forces, such as an explosion, are applied to the brain that cause significant disruption of cellular mechanics at the nanoscale [5–9]. Therefore, bTBI can result in subtle but progressive cellular and molecular changes that have the potential to produce long-term neurological degeneration and dysfunction. While the immediate consequences of bTBI are clinically silent, accumulating evidence suggests that mild trauma from blast exposure can develop post-traumatic stress disorder (PTSD) and chronic traumatic encephalopathy (CTE) [10,11], even lead to neurological changes, upper respiratory injuries, mild and moderate neurological damage, and lung damages [12]. Damages from blast injuries impact the central nervous system (CNS) (see Fig 1A) and as well as the neural progenitor cells of the CNS. Adult hippocampal progenitor cells (AHPCs) are multipotent neural cells in the adult brain. They are capable of proliferation and differentiation into neurons, oligodendrocytes, and astrocytes [13,14]. Due to their regenerative potential and multipotency, AHPCs are suitable candidates for shockwave exposure experiments aimed at investigating how bTBI [13,15] alters cellular proliferation and differentiation processes, as well as mechanical properties.

Along with the extracellular matrix, plasma membrane, transmembrane receptors, cytoskeleton, and nuclear architecture, cell type affects how cells respond to applied forces, enabling neurons and glial cells to respond to their physical surroundings. However, when the forces become too strong, as in TBI, these components may respond differently or fail to function, leading to long-term cell damage and neurodegeneration [16,17]. Previous research has made noteworthy strides in analyzing neuronal plasma membrane disruption across various injury models and revealed a positive correlation between trauma severity and the degree of membrane disruption [18]. Stem cells subjected to mechanical trauma experience significant alterations in their ability to proliferate and differentiate, underscoring the sensitivity of these cells to mechanical stimuli [19]. These mechanical forces play a critical role in modulating stem cell behavior by influencing their proliferation and differentiation [20]. It was also found that the mechanical environment and stiffness of CNS tissue or biomaterials can affect the differentiation, adhesion, and migration of stem cells. Biomaterial mechanics matching to CNS tissue is important for regenerative medicine because such mechanical cues strongly modulate cellular behavior and the success of therapeutic strategies [21]. Therefore, it is important to investigate how mechanical forces affect the nanomechanical properties of stem cells following blast trauma, which may contribute to advancements in bTBI diagnostics and treatment.

In this regard, Atomic Force Microscopy (AFM) [22,23] is a valuable tool that enables the measurement of critical factors such as Young’s modulus, deformation, viscosity, and retardation time that are key indicators of cellular behavior, health and function (Fig 1D-1E). It has been extensively used in brain cellular mechanics research to measure how trauma affects cellular structure [24]. Magdesian et al. [25] utilized AFM to demonstrate that axonal degeneration resulting from TBI and nerve compression leads to significant biomechanical changes in axons like neural damage and dysfunction. Additionally, recent advances like immunocytochemistry (ICC) techniques have enabled researchers to more precisely measure post-trauma cellular responses, particularly in relation to neural regeneration [26]. For example, ICC was used to analyze brain specimens from 11 TBI patients and showed an increased expression of neural stem/progenitor markers in the perilesional cortex [27]. Despite recent advancements, the early biomarkers of pathology that may be behind the long-term effects of bTBI are not yet fully understood. Since bTBI may lead to subtle, gradual changes in cellular and molecular mechanisms that contribute to long-term neurological dysfunction, it is critical to study these modifications at the early stage. Therefore, the goal of our study was to determine these changes by investigating cellular morphology, nanomechanical properties, and their functional implications at the single molecule level (see Fig 1E). By characterizing these parameters, we aimed to identify early indicators of damage that could be utilized in future diagnostics and interventions. This study incorporated unique experimental setup, such as subjecting live AHPC cells *in vitro*, instead of live animal models to the blast wave. These AHPCs were subjected to a shockwave generated through an extended tube from a distance, potentially allowing air blast and sound release to affect the cells. These distinctive aspects enabled a more thorough investigation of the neural cells’ responses to the applied shockwave. This integrated approach mimicked the bTBI and enabled us to explore how the changes nanomechanical, viscoelastic properties of AHPCs correlate with cellular differentiation, survival, and proliferation post shockwave exposures of two different magnitudes and two different directions, contributing to the development of therapeutic strategies and targeted diagnostics aimed at mitigating long-term damage caused by bTBI.

## Materials and methods

### Shockwave generation and propagation

A custom-built, compression-driven ‘Schedule 80’ shock tube was used to generate high-pressure, which describe the overpressure that occurs following a real-life blast that can lead to a blast-induced TBI. To make this arrangement successful, the main setup included a 3/4-inch inner diameter, a 4-inch driver, and a 30-inch driven section separated by a Dead Soft aluminum diaphragm [28], which isolated the driver from the driven region containing APHCs that were placed at the end of the driven section. Shockwaves were generated by filling the driver with compressed air until the diaphragm ruptured, sending a shockwave through the driven section (see Fig 1B). Cells were exposed under four conditions: two high blast overpressure levels—single (14.5 psi / 100 kPa for 1.76 ms, classified as mild severity) and double (29 psi / 200 kPa for 3.52 ms, classified as moderate severity) based on established blast injury severity thresholds [29], and two directions— ‘top-to-bottom’ (simulating downward-directed blast forces) and ‘bottom-to-top’ (simulating upward-directed blast forces). In the ‘top-to-bottom’ setup, the shockwave passed through the lid of the petri-dish containing the APHCs, air, cell media, and then the cells. In the bottom-to-top setup, the shockwave was applied directly to the base of the petridish for a more direct impact (Fig 1C). Since wave impedance is a function of material density and wave speed, a transition between materials with different impedance alters the energy distribution, determining the reflected and transmitted wave intensities. Due to these abrupt transitions in pressure, shockwaves can significantly influence cellular behavior and interactions. For example, in the ‘top-to-bottom’ setup, each layer: lid, air, media that introduces an impedance mismatch, reflects part of the wave, weakening the transmitted shockwave and minimizing cytoskeletal disruption. This relationship between reflection and transmission at each interface is described by Zhao et al. [30] as:


$$\begin{array}{ccc} R & = & \frac{\lambda -1}{\lambda +1}I, \end{array}$$ (1)

$$\begin{array}{ccc} T & = & \frac{2\lambda }{\lambda +1}I, \end{array}$$ (2)

$$\begin{array}{ccc} \lambda & = & \frac{\rho_{2}C_{2}}{\rho_{1}C_{1}} \end{array}$$ (3)

where λ denotes the wave impedance ratio, with $\rho_{1}$ and $\rho_{2}$ as the densities of materials 1 and 2, respectively, and *C*_1_ and *C*_2_ as the corresponding wave speeds in each material.

Multiple transitions dissipate energy at each interface, the ‘top-to-bottom’ shockwave loses strength before reaching the cells. In contrast, ‘bottom-to-top’ exposure reduces loss of energy, enhancing shear forces, and producing stronger shockwaves. Thus, impedance matching can control how much of the shockwave will be reflected or transmitted at each interface during shockwave propagation across different materials.

### Cell culture

Adult rat hippocampal progenitor cells (AHPCs) were gifted by Dr. F.H. Gage, Salk Institute, La Jolla, CA. The AHPCs were isolated from the hippocampus of adult Fisher 344 rats with prior approval from the Animal Care and Use Committee of the Salk Institute. AHPCs were cultured in a T25 tissue culture flask (Thermo Fisher Scientific) coated with poly-L-ornithine (10 µg/mL; Sigma Aldrich) overnight at room temperature, rinsed with an excess volume of phosphate buffered saline (PBS) the following day, and incubated with a laminin (10 µg/mL; Cultrex by Trevigen) solution made in PBS. AHPCs were then incubated overnight at 37° C in 5% CO_2_ incubator. The following day the laminin solution was removed, rinsed with PBS and stored frozen until use. Cells were cultured in maintenance medium (MM) composed of Dulbecco’s modified Eagle’s medium/Ham’s F-12 (DMEM/F-12, 1:1; Gibco by Thermo Fisher Scientific) supplemented with 2.5 mM GlutaMAX (Thermo Fisher Scientific), 1 x N2 supplement (Gibco by Thermo Fisher Scientific),1 x Penicillin/streptomycin (Gibco by Thermo Fisher Scientific), and 20 ng/mL basic fibroblast growth factor (bFGF; Gibco by Thermo Fisher Scientific). The cells were incubated at 37 °C in a 5% $CO_{2}$ atmosphere. AHPCs in the flask were fed with MM every 2 days by performing half media changes. They were monitored daily to ensure they were contamination-free and harvested when the cell confluency reached 80%. The multipotential nature of the AHPCs was verified by immunocytochemistry.

#### Top-to-bottom shockwave exposure experiments.

Cells were initially seeded into poly-L-ornithine/laminin (POL) coated 35 mm petridishes (Falcon) at a density of 40,000 cells/dish. Prior to seeding, cell counts were performed using Trypan Blue (Gibco) and a hemocytometer. Dishes were maintained in MM for 6 days *in vitro* (DIV) with standard half media changes. At 4 DIV, a subset of the dishes was exposed to shockwave blast injury. The cells within the dishes were cultured for an additional 48 hours at which time the cells from the dishes were harvested from the petri dishes by adding warmed 0.05% Trypsin EDTA solution (Corning) to the dishes and placing the dishes in an incubator for 2 minutes. Cells were then resuspended with MM and transferred to a 15 mL centrifuge tube and centrifuged for 6 minutes at 300 RCF. After cell pellet was formed, supernatant was removed and cells were resuspended with MM. The harvested cells were then expanded onto POL-coated coverslips at a density of 3,000 cells/coverslip. Half of the coverslips were seeded with non-exposed cells while the remaining half were seeded with cells exposed to the shockwave blast. Coverslips were cultured in MM for the first 24 hours after which time the media was switched to differentiation medium (DM; MM without the bFGF), and the cells were maintained in DM until the end of the culture period. For the single shockwave blast exposure experiments the coverslips were maintained until 13 DIV, and for the double shockwave blast exposure the coverslips were maintained until 12 DIV (**S1 Fig**).

#### Bottom-to-top shockwave exposure experiments.

Cells were initially seeded into POL-coated 35 mm petridishes at a density of 20,000 cells/dish. Dishes were maintained in MM for 6 DIV with standard half media changes. At 4 DIV, a subset of the dishes was exposed to shockwave blast injury. The cells within the dishes were cultured for an additional 48 hours at which time the cells from the dishes were harvested and expanded onto POL-coated coverslips at a density of 2,000 cells/coverslip as previously described. Half of the coverslips were seeded with non-exposed cells while the remaining half were seeded with cells exposed to the shockwave blast. Coverslips were cultured in MM for the first 24 hours and the media was switched to DM after that. The cells were maintained in DM until the end of the culture period. For both the single and double shockwave exposure experiments the coverslips were maintained until 13 DIV (**S1 Fig**).

### Atomic force microscope (AFM) setup

Nanomechanical measurements of the AHPCs were performed using an atomic force microscope (AFM) (Bruker BioScope Resolve system) integrated with a NanoScope V controller. The AFM was operated in Peak Force Quantitative Nanomechanical Mapping (PF-QNM) mode, which simultaneously provided high-resolution imaging and nanomechanical property mapping. This mode provided precise measurements of key mechanical parameters such as Young’s modulus, deformation, dissipation, and adhesion, all while minimizing the risk of damaging the delicate biological samples. Cells were cultured for 72 h under standard growth conditions to reach the desired confluency and were then exposed to single or double blast-induced shockwaves at defined pressure levels using the shock tube apparatus, as detailed in the shockwave exposure protocol. Immediately after shockwave exposure, the dish with AHPCs was placed on the AFM stage (connected to the sample heater) in a vibration isolation chamber to maintain a controlled temperature environment during measurements. Control cells were also retrieved from the incubator alongside treated cells to maintain consistency for comparison. Pre-calibrated PFQNM-LC-CAL-A probes, specifically designed for live cell applications, were employed for single-cell measurements. These probes, with a 70 nm tip radius and a nominal spring constant ranging from 0.074 N/m to 0.101 N/m, offered the necessary sensitivity and flexibility for probing soft biological materials without inducing excessive deformation. A controlled force of 300 pN was applied during each indentation, ensuring gentle yet consistent contact with the cell membrane and minimizing structural disruption. The AFM scan rate and scan size were set at 0.242 Hz and ranging from 17 μm to 50 μm, respectively, with 256x256 pixel size, achieving a balance between spatial resolution and throughput. This allowed for accurate nanomechanical mapping of each cell’s surface topography, capturing critical mechanical properties. For viscoelastic property measurements, we utilized the AFM ‘Ramp and Hold’ to maintain a constant distance between the AFM tip and the cell while tracking time-dependent force. We held the probe on the cells for 2 seconds and captured these stress relaxation curves using an MLCT-D probe in contact mode. These probes featured a tip radius of 20 nm and a spring constant of 0.03 N/m, making them suitable for capturing the time-dependent mechanical behavior of the cells. All measurements were performed under carefully controlled ambient conditions, with temperature and humidity regulated to preserve cell viability throughout the experimental process.

### Analysis for nanomechanical measurements

The nanomechanical data obtained from individual cells were processed and analyzed using Bruker NanoScope Analysis software. This software enabled a detailed examination of both the structural and mechanical properties of the cells at the nanoscale. High-resolution images and nanomechanical maps were exported as .spm files, providing comprehensive visualization of cell surface topography and mechanical characteristics. Force curves were captured as .pfc files at 10 random locations on each cell and analyzed using the Hertz model [31,32] with a spherical indenter, yielding average Young’s modulus values for 20 cells to assess their stiffness and elastic properties. In the Hertz model, the indentation force is written as follows:


$$\begin{array}{c} F=\left(\frac{4}{3}\right)\left(\frac{E}{\left(1-\nu^{2}\right)}\right)\sqrt{R}\delta^{\frac{3}{2}}, \end{array}$$ (4)

Indentation force *F*, Young’s modulus *E*, Poisson ratio ν, indentation δ, and *R* representing the radius of the indenter are the parameters included in the Hertz model.

To characterize viscoelastic behavior, time-dependent force curves were obtained by maintaining a fixed position of the height sensor for two seconds during each measurement. The resulting stress relaxation curves were analyzed using Kohlrausch-Williams-Watts (KWW) models [33] to extract parameters such as retardation time and viscosity described by the following expression:


$$\begin{array}{c} \sigma_{\text{rel}}(t)=\sigma_{0}\left[\exp\left(-{\left(\frac{t}{\eta }\right)}^{\beta }\right)\right]+\sigma_{f} \end{array}$$ (5)

The final stress is represented by $\sigma_{f}$ as time (*t*) goes towards infinity. Likewise, $\sigma_{0}$ is the func*t*ion representing stress which is dependent on *t*. The shape parameter (β) and characteristic life (η) have relationships with the time *t*hat the load has been applied to. These metrics characterize the viscoelastic behavior of the material and give insight into how the stress develops when the load has been applied for a very long period of time. A total of 40 data points per cell type were analyzed, offering a thorough assessment of the viscoelastic properties of the cells. Representative stress relaxation curves for each condition are shown in Fig 4, illustrating the distinct mechanical responses of the different cell lines.

### Propidium iodide (PI) staining

Cell viability was determined using propidium iodide (PI; Sigma Aldrich) to stain dead cells within the cultures. For the ‘top-to-bottom’ shockwave exposure experiments, PI staining was performed at 5 days *in vitro* (DIV) (24 hours after shockwave blast injury) and at the end of the culture period (**S1 Fig** A-B). For the ‘bottom-to-top’ shockwave exposure experiments, PI staining was performed within 6 hours following shockwave exposure and at the end of the culture period (**S1 Fig** C). The PI was diluted to 1.5 µM in cell culture medium. The cells were incubated in PI solution for 20 minutes at 37°C in 5% CO_2_. As a reagent control, one coverslip with cells was incubated with 70% ethanol for 5 minutes to intentionally kill all the cells prior to the addition of the PI solution. After the 20-minute incubation, the cells were rinsed with ice-cold 0.1 M PO_4_ buffer for one minute and then were fixed with 4% paraformaldehyde (PFA, Thermo Fisher Scientific) made in 0.1 M PO_4_ for 20 minutes. Cells were then rinsed three times for 7 minutes each with phosphate-buffered saline (PBS) to remove all the fixative and then incubated with 4^′^,6-diamidino-2-phenylindole (DAPI; 1:500; Invitrogen by Thermo Fisher Scientific) diluted in PBS for 60 minutes at room temperature in the dark. The cells were then rinsed four times for 8 minutes each with PBS. Coverslips were then mounted onto microscope slides with Fluoromount-G with DAPI (Invitrogen by Thermo Fisher Scientific). Propidium iodide staining was used to evaluate cell survival. As a positive control for the PI reagent, samples with AHPCs were incubated with 70% ethanol, a condition known to kill most cells, resulting in a high percentage of PI-labeled cells. In all previous studies where we have used PI staining, this reagent control has verified that the PI staining was working properly.

### Immunocytochemistry (ICC)

At the end of the culture period, immunocytochemistry was used to evaluate the extent of proliferation and differentiation of the AHPCs following shockwave exposure (**S1 Fig**). Briefly, the AHPCs on the coverslips were rinsed with ice-cold 0.1 M PO_4_ buffer for one minute and then incubated in 4% paraformaldehyde (PFA) made in 0.1 M PO_4_ buffer for 20 minutes. Following fixation, the coverslips were rinsed three times for 7 minutes each with phosphate-buffered saline (PBS) to remove all the fixatives. The coverslips were then incubated in a blocking solution composed of 0.2% Triton X-100 (Thermo Fisher Scientific), 5% normal donkey serum (NDS) (Jackson ImmunoResearch), and 0.4% bovine serum albumin (BSA) (Sigma-Aldrich) in PBS at room temperature for 90 minutes. Primary antibodies—Rabbit α Ki-67 (1:300, IgG; Abcam), Mouse α TuJ1 (1:200, IgG; R&D Systems), Mouse α MAP2ab (1:200, IgG; Sigma-Aldrich), Mouse α RIP (1:200, IgG; DSHB, Iowa City, IA), and Mouse α GFAP (1:200, IgG; Sigma-Aldrich)—were diluted in the blocking solution. See **S1 Table** for details on cell markers these primary antibodies are being used to indentify. The cells were incubated in the primary antibody solution overnight at 4° C. The next day, the samples were rinsed four times for 8 minutes each with PBS. To prepare the secondary antibody solution, Donkey α Rabbit AF488 (1:300, IgG; Jackson ImmunoResearch), Donkey α Mouse AF488 (1:200, IgG; Jackson ImmunoResearch), and Donkey α Mouse Cy3 (1:200, IgG; Jackson ImmunoResearch) were diluted using the blocking solution, which also contained DAPI (1:500). The cells were incubated with the secondary antibody solution at room temperature in the dark for 90 minutes and then were rinsed with PBS four times for 8 minutes each. Coverslips were then mounted onto microscope slides with Fluoromount-G with DAPI. Notably, the DAPI counterstaining used throughout the results section was performed on paraformaldehyde fixed cells. The DAPI was not used as a live cell marker. The total number of cells in an image field was determined by counting the number of DAPI stained nuclei.

### Leica fluorescent microscope

For cells cultured in 35 mm dishes, the cells were imaged using an inverted Leica fluorescent microscope (Leica DMI4000B; Leica Microsystems) equipped with standard epifluorescence illumination and a Leica DFC310 FX (Leica Microsystems) digital camera. For cells cultured on coverslips, the cells were imaged using an upright Leica fluorescent microscope (Leica DM5000B; Leica Microsystems) equipped with standard epifluorescence illumination and a Q Imaging Retiga 2000R (Q Imaging) digital camera. A 20× objective was used to obtain images for quantitative data analysis, while a 40× objective was used to obtain high magnification images of cellular morphology.

### Data acquisition and statistical analysis

The images of the AHPCs were analyzed and quantified using ImageJ software (http://imagej.nih.gov/ij). PI, Ki-67, TuJ1, MAP2ab, and RIP immunoreactive cells were counted using the Cell Counter tool in ImageJ, and the percentage of immunoreactive cells was determined as the number of positively labeled cells divided by the number of DAPI-labeled nuclei. For each independent experiment, ten image fields were acquired for each stain or antibody. Consequently, 20 or 30 image fields per condition were quantified from N = 2 or N = 3 independent experiments, respectively. Then, the percentage of immunoreactive cells was averaged across all these image fields, resulting in one representative measurement per experimental condition. On the other hand, a total of 20 individually indented cells per condition were quantified for the AFM analysis. The control group comprised cells from N = 2 independent experiments, while the shockwave-exposed group comprised cells from N = 3 independent experiments. All means are reported with standard deviation (mean ± SD) or standard error of the mean (mean ± SEM). Graph Pad Prism 10 (Graph Pad Software, Inc., San Diego, CA) was used for ICC/PI, and OriginPro 2022 (64-bit) SR1 version 9.9.0.225 (Academic) was used for AFM statistical analysis and graph-making. Statistical comparisons were conducted on the mean of these replicates (N = 2 or N = 3 replicates per condition), using either an unpaired T-test with Welch’s correction (two-group comparisons) or ordinary one-way ANOVA using Tukey’s multiple comparisons test, with α=0.05 for both ICC/PI and AFM. The significance levels are denoted as follows: * for *P* < 0.05; ** for *P* < 0.01; *** for *P* < 0.001; and **** for *P* < 0.0001.

The units used for statistical analysis in this study vary depending on the assays and their designs. For example, in the case of the population-averaged parameters (ICC, PI), the unit of analysis is an image field from an independently prepared coverslip, while cells within an image field act as technical sub-samples. In contrast, for ‘single-cell AFM nanoindentation’ experiments, cells are analyzed individually because intercellular mechanical heterogeneity is a well-known factor in this type of measurement [34–36]. In compliance with the standard approach in single-cell AFM experiments [37–39], we therefore used the individual cell, pooled across N = 3 independent preparations, as the statistical unit for the AFM comparisons.

## Results

### Morphological changes in cells following shockwave exposures

**Fig 2:**
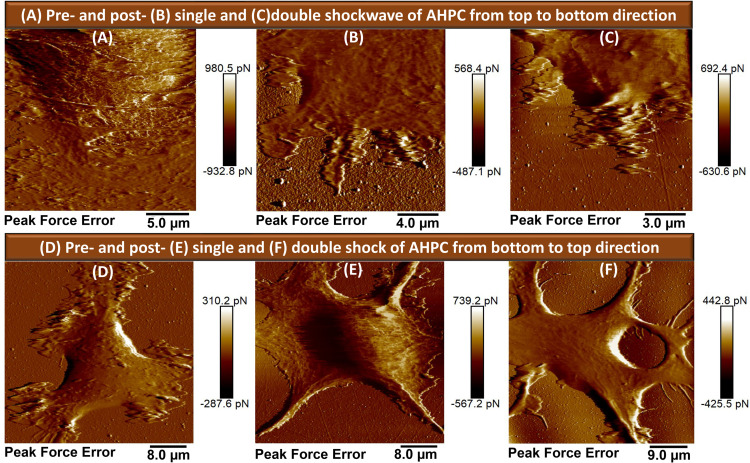
Peak force error images of cells obtained using AFM under different shockwave exposure conditions. The top section shows images of control cells(A) alongside cells exposed to a shockwave from the top at 14.5 psi(B) and 29 psi(C). The bottom section presents control images(D) along with images of cells exposed to a shockwave from the bottom of the cell dish at 14.5 psi(E) and 29 psi(F). This comparison highlights morphological changes in cell surfaces due to directional shockwave exposure at varying pressures.

**Fig 3:**
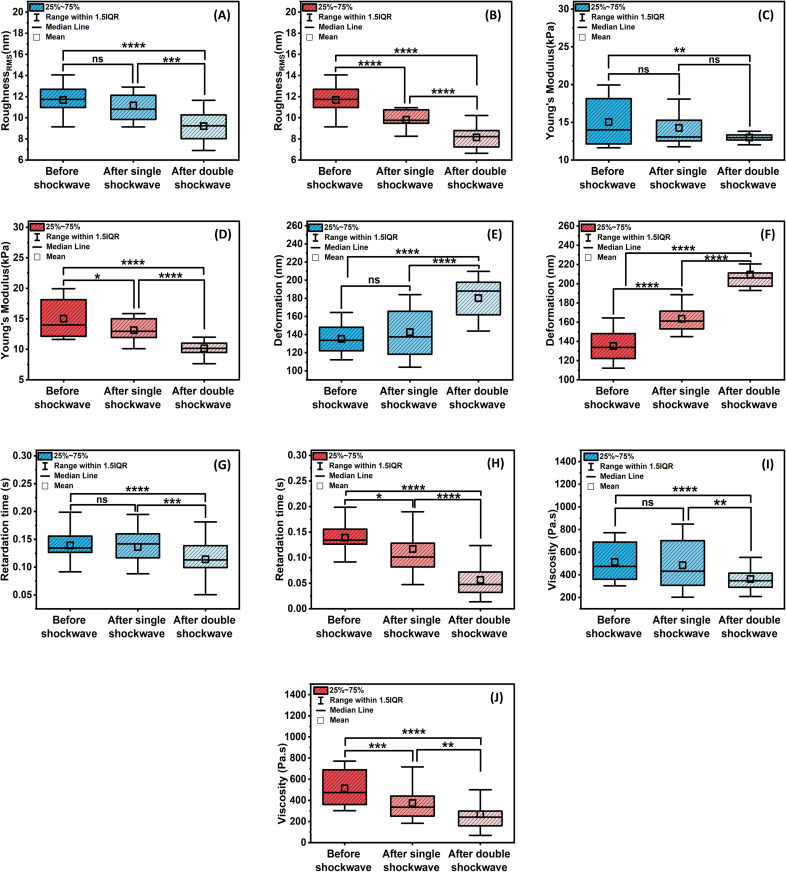
Effects of shockwave exposure from top-to-bottom (A, C, E, G, I) and bottom-to-top (B, D, F, H, J) directions on the morphological and mechanical properties of AHPCs. Box plots illustrate comparisons across three conditions: before shockwave (cells were not exposed to shockwaves), after single shockwave, and after double shockwave exposure: under top-to-bottom shockwave exposure for (A) surface roughness, (C) Young’s modulus, (E) deformation, (G) retardation time, and (I) viscosity. Similarly, the impacts of single and double shockwave exposure from the bottom-to-top direction on AHPCs are shown in box plots for (B) surface roughness, (D) Young’s modulus, (F) deformation, (H) retardation time, and (J) viscosity. Significant decreases in roughness, Young’s modulus, viscosity, and retardation time, along with an increase in deformation, are observed after shockwave exposure, particularly following double shockwave exposure in both cases. ‘X——X’ represents the upper and lower extremes of the data, while the error bars extend to a range of 1.5 times the interquartile range (IQR). The mean is depicted by the small box, and the median is indicated by the black line. Statistical significance is denoted as follows: ns (not significant), * *p* < 0.05, ** *p* < 0.01, *** *p* < 0.001, **** *p* < 0.0001.

We investigated the morphological changes of AHPC cells subjected to shockwaves with varying intensities and orientations. In control cells (that were not exposed to shockwaves), the actin cytoskeleton was strongly assembled into actin-rich structures with clear assemblies and defined striations contributing to the mechanical strength, and structural integrity of the cell (see Fig 2A,2D). These stress fibers are mainly composed of actin and myosin and are connected to focal adhesion sites on the plasma membrane [40] that ensure mechanical resilience and stable substrate adhesion. Upon shockwave exposure, this cohesive and dense network started becoming weaker and aggregated (Fig 2B-2C) primarily around the cell periphery area. Notably, actin filaments became smoothened and also retracted from the perinuclear area towards the edge (Fig 2E-2F) creating an uneven cytoskeletal distribution. The retracted cells showed a rearrangement of their actin filaments into elongated fibers in the filopodia [41]. Moreover, when shockwaves were delivered from the bottom of the dish, cells displayed membrane blebbing and became more globular and even at forces <100pN. Over time, treated cells became more round in shape with surface blebbing but began restoring their shape and cytoskeletal structure around 3 hours post-exposure when they were kept in the incubator, indicating recovery (**S8 Fig**). In the further study, surface roughness analysis revealed that ‘top-to-bottom’ single shockwaves caused minimal reduction in membrane roughness (RMS: 11.17 ± 1.17 nm) compared to controls (RMS: 11.69 ± 1.69 nm). However, increased shockwave with double magnitude led to a marked decrease in roughness (RMS: 9.21 ± 1.44 nm, in Fig 3A). On the other hand, ‘bottom-to-top’ shockwaves caused further smoothening of cell surface, with roughness values of 9.83 ± 1.10 nm (single) and 8.14 ± 1.08 nm (double exposure), as shown in Fig 3B. These findings suggested that shockwave exposures at higher magnitudes/ intensities and ‘bottom-to-top’ direction weaken the actin cytoskeleton, leading to reduced membrane roughness and altered cell morphology.

### Influence of shockwave application on cell mechanics

#### Cellular stiffness and deformation.

Since bTBI is a result of the interaction between external physical forces and the biomechanical specificity of the brain, it is crucial to understand the cellular mechanical responses such as stress and strain that define tissue mechanics [42,43] and their behavior. Therefore, we captured a detailed mapping of the nanomechanical properties after immediate shockwave exposure. We fit the captured force-distance curves with the Hertz model (see Eq. 4 in section **Analysis for Nanomechanical Measurements**) to obtain the changes in Young’s modulus. Our result showed that cellular stiffness or Young’s modulus of the controlled cells was 15.02 ± 3.17 kPa. After ‘top-to-bottom’ shockwave exposure, it decreased to 14.32 ± 2.80 kPa for single exposure and further dropped to 12.98 ± 1.20 kPa after double shockwave exposure (Fig 3C). Simultaneously, the corresponding cell deformation was analyzed. Control cells exhibited an average deformation of 135.23 ± 17.20 nm, increasing to 142.65 ± 27.34 nm after single exposure and 180.18 ± 21.14 nm after double shockwave exposure (see Fig 3E). Furthermore, the direct mechanical effect of the ‘bottom-to-top’ configuration was studied (**Shockwave Generation and Propagation**). In this set-up, single shockwave exposure was found to be sufficient to cause a detectable change in cell stiffness, with Young’s modulus of 13.14 ± 3.16 kPa, and it further decreased to 10.15 ± 1.12 kPa, as depicted in Fig 3D. Similarly, the deformation results followed a comparable trend; a single shockwave exposure yielded an average of 163.45 ± 14.17 nm, whereas double shockwave exposure resulted in a higher average value of 209.48 ± 19.32 nm, as shown in Fig 3F. In short, these results show that shockwave exposure leads to a dose-dependent decrease of Young’s modulus and an increase in cellular deformation in AHPCs with ‘bottom-to-top’ shockwaves, leading to a significant decrease in stiffness and increase in deformation compared to ‘top-to-bottom’ exposure, showing orientation-dependent mechanical effects.

#### Viscoelastic properties.

**Fig 4:**
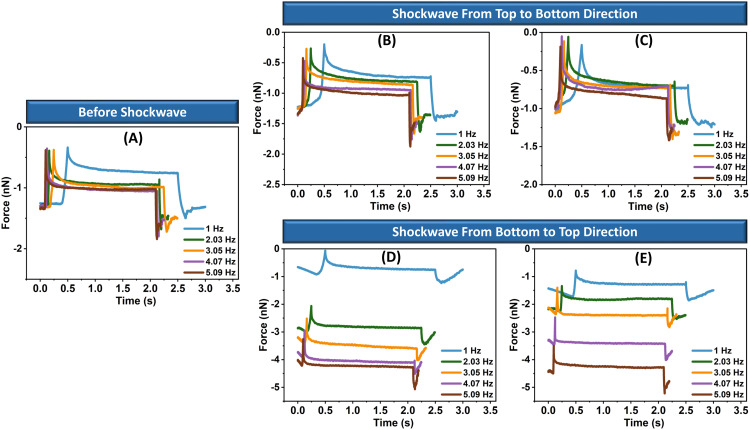
Representation of force-time curve measured in AHPCs before shockwave exposure (A), after a single shockwave (B, D), and after double shockwave exposures (C, E) from both top-to-bottom (B, C) and bottom-to-top (D, E) directions, with the scan rate ranging from 1 Hz to 5.09 Hz. The tip approached and detected the cell surface, and during the 2-second holding period, the loading force began to decrease because of the cell’s viscoelastic properties.

Similarly, we investigated the relaxation behavior of AHPCs to understand how different bTBI conditions influence viscoelastic properties that were obtained at varied scan rates and loading velocities. We used ‘Ramp and Hold’ to characterize the cellular surface viscoelasticity by studying their dynamic stress response as a function of applied strain. The AFM tip was approached towards the cell surface at scan rates of 1 Hz, 2.03 Hz, 3.05 Hz, 4.07 Hz, and 5.09 Hz, with corresponding loading velocities of 10 μm/s, 20.3 μm/s, 30.5 μm/s, 40.7 μm/s, and 50.9 μm/s, respectively. The AFM tip approached the cell during each measurement until a defined loading force threshold was reached. Then, the cantilever base displacement constant was held for 2 seconds before retracting at the same velocity. These time-series force relaxation curves in Fig 4A-4E showed a nonlinear decrease in force after reaching the set point, characteristic of viscoelastic material behavior. At lower scan rates (e.g., 1 Hz), the cell surface showed a slower and more extended force decay since cells had more time to respond to the applied stress. In contrast, at higher scan rates (e.g., 5.09 Hz), the force decayed more steeply and rapidly, indicating a stiffer response due to limited time for viscous deformation due to the fast movement of the probe. However, compared to the controlled AHPCs’ behavior, the relaxation process was similar across all cases (Fig 4A-4E), whether the shockwave was applied from top-to-bottom (Fig 4B-4C) or bottom-to-top (Fig 4D-4E), regardless of the exposure duration of 1.76 ms or consecutive two exposures of 1.76 ms each.

We employed the Kohlrausch-Williams-Watts (KWW) function [33] (see Eq. 5 in section **Analysis for Nanomechanical Measurements**, an empirical model to describe the dispersion processes in viscoelastic systems to assess the relaxation behavior quantitatively. The relaxation time (τ) for control cells was determined to be 0.139±0.03s. Following a single shockwave exposure (top-to-bottom), the relaxation time showed minimal change at 0.136±0.03s, whereas double shock exposure resulted in a decrease to 0.114±0.03s (see Fig 3G). In contrast, the bottom-to-top shockwave exposure produced more significant effects: single shockwave measurements yielded a relaxation time of 0.117±0.09s, and double shockwave reduced those times even more to 00.056±0.03s (Fig 3H) respectively. The analysis of cellular viscosity also revealed notable trends. Control cells exhibited a viscosity of 512.98±163.95Pa·s. After a top-to-bottom single shockwave exposure, the viscosity decreased to 484.49±209.75Pa·s, with a further reduction to 362.46±95.63Pa·s respectively following double shock exposure (Fig 3I). For cells exposed to bottom-to-top shocks, the viscosity following a single shock was 374.22±149.01Pa·s, and this value dropped significantly to 268.61±164.33Pa·s respectively after a double shock (Fig 3J). Overall, our results show that although AHPCs’ viscoelastic relaxation behavior was consistent across bTBI conditions, exposure to ‘bottom-to-top’ shockwaves resulted in more noticeable decreases in viscosity and relaxation time, suggesting a higher level of cellular dissipative mechanics impairment.

### Assessment of AHPC viability after shockwave exposure

To evaluate whether AHPCs remain sufficiently viable after exposure to shockwaves, we performed viability assessments using propidium iodide (PI) staining at various time points post-exposure. The PI staining performed 24 hours after shockwave exposure showed that exposure to single or double shockwave blast had little to no effect on cell viability shortly after shockwave compared to the control group as the cell viability remained above 99% for all conditions (**S2 Fig**). However, there was a slight, yet significant, decrease in the percentage of viable cells for the double shockwave exposed samples compared to the single shockwave samples, though both groups remained above 99% (**S4 Fig** A). Furthermore, cell viability remained high even at the end of the culture period, with the percentage of PI-labeled cells for both shockwave groups at 13 DIV above 99% for all culture conditions (**S3 Fig**). As with the early time point, there was a slight, yet significant decrease in cell viability for the double shockwave conditions compared to the single shockwave condition, though both groups remained above 98% (**S4 Fig** B). These results indicated that there was a negligible effect of single or double shockwave exposures from ‘top-to-bottom’ direction on the viability of the AHPCs shortly after exposure or after a longer-term culture.

To reduce the effects of shockwaves traveling through the dish lid, air, and culture media, the shockwave direction was inverted to expose the cells from the ‘bottom-to-top’ direction for a more direct impact. Cell viability was assessed 3 hours after shockwave exposure to evaluate its short-term impacts. Regarding the single shockwave condition, the viability percentages were above 99% for both the control and shockwave-exposed samples (**S5 Fig** A-C). When comparing the control and double shockwave-exposed samples, the viable cell percentages were both above 98% (**S5 Fig** D-F). Cell viability was reassessed after 10 DIV to ensure viability at the time of the ICC. Cell viability was high (above 99%) for both the single and double shockwave-exposed AHPCs, confirming the cells’ high viability post-expansion (**S6 Fig** A-F). With respect to both time periods, results showed that single and double shockwave exposures from the ‘bottom-to-top’ direction had minimal impact on cell survival, with no significant differences in viability compared to their non-exposed controls. Comparing the cell viability results of the single and double shockwave-exposed samples, viability decreased slightly following double shockwave exposure at the 4 DIV, though there were no significant differences between the shockwave conditions at both time periods (**S7 Fig** A-B). This suggests shockwave exposure at these strengths had little effect on the viability of remaining adherent AHPCs.

### Impact of shockwave exposure on AHPC differentiation potential

**Fig 5:**
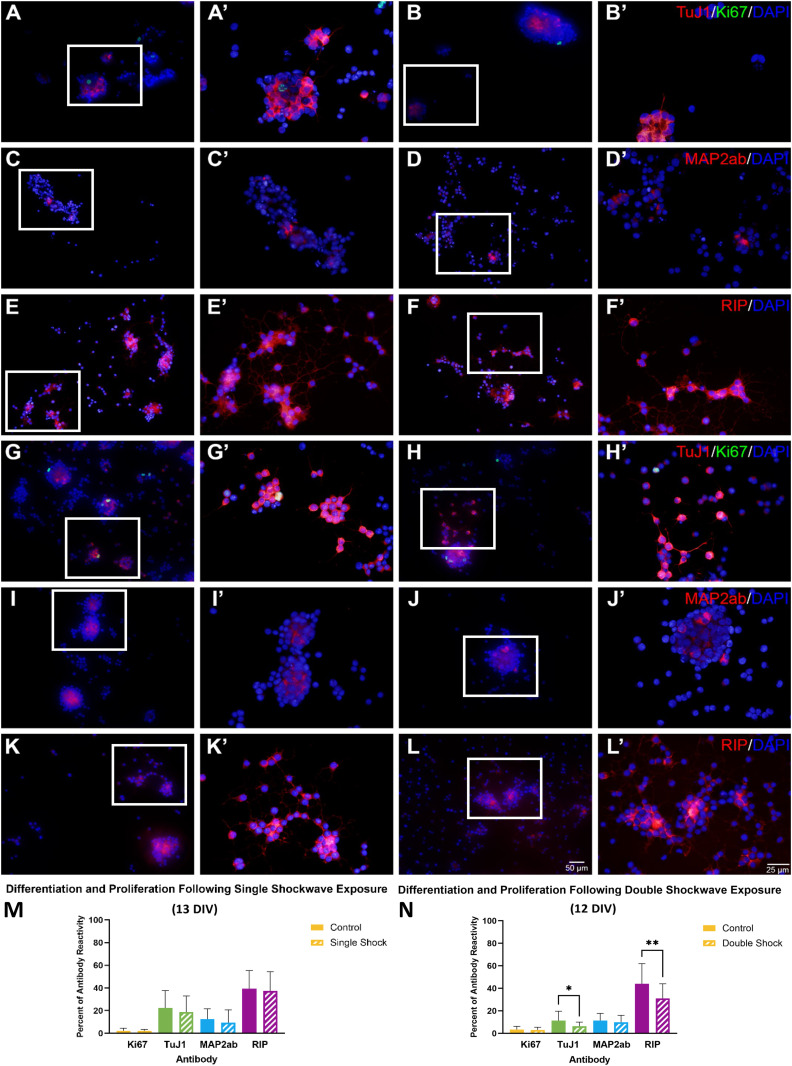
Differentiation and proliferation of AHPCs at 12-13 DIV following single or double shockwave exposure from top-to-bottom. Fluorescence images of AHPCs on coverslips at 12 or 13 days in vitro (DIV) following single (A-F’) or double (G-L’) shockwave exposure from above. Cells were single or double immunostained with an immature neuron marker (TuJ1, red; A-B’), a cell proliferation marker (Ki67, green; A-B’), a maturing neuronal marker (MAP2ab, red; C-D’), an oligodendrocyte marker (RIP, red; E-F’), and a cell nuclei marker (DAPI, blue; A-F’). Images A-F were taken using a 20x objective, with a scale bar of 50 µm. Images A’-L’ represent the boxed region in each respective image at a higher magnification, with a scale bar of 25 µm. M: There was no significant difference between the culture conditions. Bars represent the mean percentage of immunolabeled cells, and the error bars represent the standard deviation (± SD). N = 2 independent experiments, with 20 image fields quantified for each condition. N: There was a significant decrease in the expression of TuJ1 and RIP following double shockwave exposure. There was no significant difference between the culture conditions for the remaining antibody labeling. Bars represent the mean percentage of antibody-labeled cells, and the error bars represent the standard error of the mean (±SEM). N = 3 independent experiments; 30 image fields were quantified for each condition.

To evaluate the impact of shockwave exposure on the differentiation and proliferation potential of AHPCs, immunocytochemical analysis was conducted to assess the expression of neuronal, oligodendrocyte, astrocyte, and proliferative markers across all experimental conditions. The results showed there were no significant differences in the expression of any of the antibodies tested between the control and single shockwave exposed samples; however, significant decreases in antibody expression began to be seen following double shockwave exposure (see Fig 5). The percentage of TuJ1-expressing developing neurons was 22.29 ± 15.48% compared to 18.84 ± 14.03% for the control and single shockwave exposure conditions, respectively (Fig 5A-5B’,M). In contrast, there were significant changes following double shockwave exposure as illustrated by the significant decrease in TuJ1-expression, where the percentage decreased from 11.41 ± 1.7% for the control condition to 6.42 ± 0.7% for the double shockwave condition. The percentage of TuJ1 expression following double shockwave exposure was also significantly lower than that seen following single shockwave exposure (Fig 5G-5H’, N, and **S4 Fig** C). MAP2ab expression showed a minimal decrease following shockwave exposure with expression dropping from 12.54 ± 9.15% to 9.28 ± 11.48% for single shockwave exposure condition and from 11.49 ± 1.3% to 10 ± 1.3% for double shockwave exposure conditions (Fig 5C-5D’, I-J’,M-N). RIP expression slightly decreased, going from 39.4 ± 16.04% for the control condition to 37.29 ± 17.21% for the single shockwave condition (Fig 5E-5F’,M) but showed a significant decrease in expression following double shockwave exposure with the average percentage of RIP-expressing cells dropping from 44.06 ± 3.3% to 31 ± 2.4% (Fig 5K-5L’,N). There was no GFAP expression seen for either the control or shockwave exposed samples. Minimal change was seen in Ki67-expressing proliferating cells, (Fig 5A-5B’,G-H’,M-N). Overall, these results show that single shockwave exposure from ‘top-to-bottom’ direction had little to no effect on cell viability, proliferation, or differentiation while the double shockwave exposure had some significant effect on immature neuron and oligodendrocyte differentiation.

**Fig 6:**
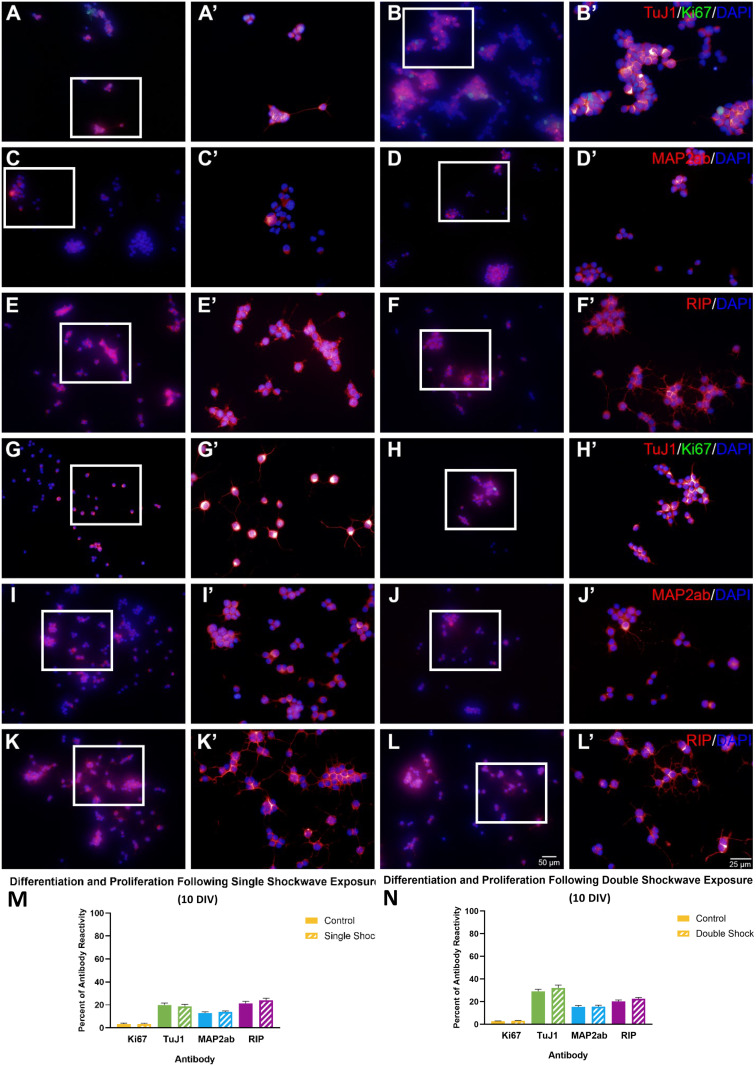
Differentiation and Proliferation of AHPCs at 10 DIV Following Single or Double Shockwave Exposure from Bottom-to-Top. Fluorescence images of AHPCs on coverslips at 10 DIV following single (A-F’) or double (G-L’) shockwave exposure from below. Cells were single or double immunostained with an immature neuron marker (TuJ1, red; A-B’), a cell proliferation marker (Ki67, green; A-B’), a maturing neuronal marker (MAP2ab, red; C-D’), an oligodendrocyte marker (RIP, red; E-F’), and a cell nuclei marker (DAPI, blue; A-F’). Images A-F were taken using a 20x objective, with a scale bar of 50 µm. Images A’-L’ represent the boxed region in each respective image at a higher magnification, with a scale bar of 25 µm. M:There was no significant difference between the culture conditions. N: There was no significant difference between the culture conditions. Bars represent the mean percentage of immunolabeled cells, and the error bars represent the standard error of the mean (±SEM). N = 3 independent experiments; 30 image fields were quantified for each condition.

However, at 10 DIV following bottom-to-top shockwave exposure, single shockwave exposure showed no consistent trend in antibody expression between control and exposed samples. Double shockwave exposure slightly increased antibody expression, but there were no significant differences between control and shockwave-exposed samples or between shockwave conditions, except TuJ1. TuJ1 expression showed no patterns or significant difference between the controls and single and double shockwave-exposed samples (20.05 ± 1.6% to 18.83 ± 1.8% and 29.12 ± 1.8% to 32.01 ± 2.6%, respectively) (Fig 6A-6B’, G-H’, M, N). Although in comparing single and double shockwave exposures, double shockwave resulted in significantly higher TuJ1 expression (32.01 ± 2.6% versus 18.83 ± 1.8%, respectively) (**S7 Fig** C). MAP2ab expression demonstrated a consistent, though non-significant, increase from the controls to the shockwave-exposed samples. Expression increased from 12.85 ± 1.1% to 13.79 ± 0.8% following a single shock and 15.4 ± 1.2% to 15.61 ± 1.2% following a double shock (Fig 6C-6D’, I-J’, M, N). No significant difference was found in a comparison of the expression between single and double shockwave-exposed samples (13.79 ± 0.8% and 15.61 ± 1.2%, respectively) (**S7 Fig** C). Expression of the RIP antibody also demonstrated consistent, though non-significant, increase from the controls to the shockwave-exposed samples. Following single and double shockwave exposures, that expression increased from 21.21 ± 1.19% to 24.17 ± 1.8%, and 20.18 ± 1.3% to 22.52 ± 1.2%, respectively (Fig 6E-6F’, K’-L’, M, N). Between the single and double shockwave exposures, expression decreased from 24.17 ± 1.8% to 22.52 ± 1.2%, which did not indicate a significant difference (**S7 Fig** C). No GFAP-immunoreactive AHPCs were detected between any of the conditions. Lastly, we selected to use Ki67 immunolabeling with the goal to determine whether cells are generally proliferative versus quiescent following the various shockwave treatments. Since Ki67 is an endogenous nuclear protein expressed during all active phases of the cell cycle (G1, S, G2, and M), while it is absent in quiescent (G0) cells, this approach was useful for estimating the overall growth fraction of the population of cells. Ki67 expression slightly decreased following single shockwave exposure from 3.38 ± 0.7% to 3.25 ± 0.6% but increased following double shockwave exposure from 2.69 ± 0.3% to 3.1 ± 0.3% (Fig 6A-6B’, G-H’, M, N). There were no significant differences between the control samples and shockwave-exposed samples, nor between the single and double shockwave-exposed samples (3.25 ± 0.6% to 3.1 ± 0.3%, respectively) (**S7 Fig** C).

## Discussion

Blast-induced trauma is strongly influenced by the presence of intermediate protective barriers between the source of the blast and the target cell, which is the underlying nervous tissue. In reality, particularly in a combat situation, the possibility that the pressure wave from the explosion will be in direct contact with nervous tissue is extremely low, as it travels along various layers of protection such as helmets, padding systems, skull bone, cerebrospinal fluid, and soft tissue. Each of these materials has unique mechanical impedance properties that can either reflect, refract, attenuate, or focus stress waves incident upon it, depending upon their intensities, before they reach neural cells. It has been shown that the directionality of the blast wave compared to anatomical geometry makes a significant contribution to the intracranial pressure distribution, shear stress localization, and tissue deformation patterns [44–46]. Thus, overhead explosions or airborne detonations may undergo a different pattern of reflection and redistribution by helmet shells and skull curvature compared to the ground-based improvised explosion that can transmit energy more directly through the cervical and basal cranial structures.These direction-related cell responses can be understood on the basis of existing biomechanical research, which shows that the shape of the skull is able to control both the direction and focus of mechanical force transmission. Biomechanically, the skull base is mechanically stronger than the skull vault, needing significantly more force and energy to break it, and loading occurs primarily axially rather than radially (for example, via the cervical spine and foramen magnum) [47]. Also, it was demonstrated experimentally that regional bone thickness variations within the skull actively influence the directions of intracranial force vectors, thus resulting in the specific neuropathological patterns that depend on the mechanical load direction [48]. Clinical data show that the direction of force application relative to skull anatomy alone affects the degree of brain damage [49]. Additionally, while protective equipment is intended to lessen the severity of the maximum injuries, the presence of multiple layers does not act to reduce the impact of the blast in a consistent manner. Instead, depending on the severity of the blast and the direction, protective equipment can produce inadvertent stress concentrations, as well as the effect of wave focusing, within the skull. Computer simulations and other research have indicated the use of helmets can alter the shape of the blast wave, which can decrease the maximum pressure while increasing the impulse or internal heterogeneity of stress [44,46].

In this regard, our comprehensive investigation simulated blast waves in a controlled laboratory setting and examined the biomechanical and developmental responses of AHPCs after exposure to mechanical shock waves of different magnitudes and directions. Since these shockwaves can be generated and transmitted through various media, their impact on cells can vary significantly depending on the propagation path due to impedance mismatching [30] and energy dissipation [50] at each interface. To assess whether multilayer transmission attenuates the intensity of the shockwave for the cells and thereby reduces its cellular effects, we built a custom compression-driven shock tube to mimic the blast wave and systematically varied the shockwave under four conditions in our experiments. While our in vitro system does not include a cranium and cannot be used as an analogy to these force transmission mechanisms, such an anatomical background suggests that *in vivo* similar variations in force direction related to our ‘top-to-bottom’ and ‘bottom-to-top’ exposures can be additionally influenced and even amplified/buffered by the mechanical properties of the skull. By combining AFM for nanomechanical analysis and ICC for the evaluation of cellular phenotypes, we provided a multidimensional perspective on how shockwaves affect the structure, function, mechanics and differentiation of AHPCs.

Firstly, the architectural findings of the AHPCs demonstrated that the actin cytoskeleton in control AHPC cells was highly organized, with stress fibers forming structural support through focal adhesions [40]. However, with increasing shockwave magnitude/ intensity, actin networks demonstrated peripheral aggregation, reduced striations, and cytoskeletal redistribution. It indicates mechanical destabilization, particularly in the double shockwave with a ‘bottom-to-top’ direction. A previous *in vivo* study demonstrated that extracellular glutamate levels increase following TBI [51,52]. The ion channels, including the NMDA receptors also interact with the cytoskeleton and membrane lipids, exhibiting mechanosensitivity directly or indirectly through conformational changes caused by force itself [53,54]. Similar to our work, Moosavi-Nejad et al. [55] also reported cytoskeletal disruptions, with focused shock wave treatment causing severe disorganization of the actin and tubulin networks in renal carcinoma cells. Numerous prior studies [32,56,57] have established a strong correlation between actin cytoskeleton remodeling and AFM-measured mechanical properties, where alterations in actin filament density, alignment, and crosslinking directly reflect alterations in cellular stiffness and surface microtopography. Beyond changes in cell morphology, the observed reduction in cell membrane roughness at higher shockwave magnitudes and in the reverse orientation further supports the hypothesis of cytoskeletal weakening. Also, these ion channels are mechanically modulated and allow the regulation of cellular responses to stress and pressure that may influence changes in the plasma membrane post-bTBI [58]. Thus, the AFM images and cell membrane surface data presented in this study provide indirect yet functionally relevant evidence of actin cytoskeletal reorganization following blast exposure. Since cytoskeletal integrity and membrane roughness are interlinked, it proves that a reduction in cell surface roughness post bTBI is directly connected with the resulting cytoskeletal damage [55].

Subsequently, shockwaves result in a significant decrease in Young’s modulus that represents a significant loss of cellular stiffness and mechanical integrity. These cells with such exposure conditions are also more deformable, as corroborated by higher cellular strain and morphological distortion. These effects are more evident with double shockwave exposure in the ‘bottom-to-top’ direction. Moreover, viscoelastic properties can reveal how cells dissipate mechanical energy and resist deformation over time, and these are critical metrics for their survival in dynamic microenvironments. Therefore, to examine whether cells can retain the characteristic viscoelastic properties of brain tissue following exposure to bTBI, we investigated cellular responses under varying mechanical loading. Our results highlighted that cells were able to hold the dual mechanical nature of brain tissue and its cellular constituents: exhibiting predominantly elastic behavior under rapid stress application and viscous behavior under prolonged or slow stress in all conditions [59]. This observation aligns with prior findings showing that injury-induced softening is typically accompanied by decreased viscosity and faster stress relaxation, reflecting cytoskeletal disassembly and impaired intracellular damping [40,55]. Additionally, our observation of the decrease in relaxation time and viscosity suggests that double shockwave exposures, regardless of direction of exposure, induce substantial softening and adaptive viscoelastic responses in the cells. Such mechanical responses have been similarly reported in [60], that explored cortical impacts in mice and showed that the shear stiffness of the injured area of the brain was significantly reduced at the time of injury and remained so for days. Moreover, the fact that shockwaves propagate through the brain so fast, affects cellular and subcellular structures, causing modification of mechanical properties, loss of elasticity and misalignment in axons, as confirmed by cortical impact and stretch injury studies [60,61]. The relatively more severe cellular mechanical instability in response to repetitive shockwave exposure in our model system fits into the general theory of cumulative mechanical vulnerability associated with traumatic brain injury. At the sub-cellular level, a single non-failing sub-threshold mechanical stress can momentarily impair the plasma membrane and cortical cytoskeleton but fails to lead to overt damage due to the ability of the cell to repair itself [62]. The second stress applied before the completion of the repair process causes much greater cytoskeletal impairment compared to one application of stress of similar intensity, as shown in an axon stretch-injury model in which repetitive very mild stretching enhanced growth cone collapse and cytoskeletal mislocalization beyond what was seen after one mild stretch [63]. Similar cumulative damage has been noted in shear-sensitive blood vessels exposed to repetitive blast. This intracellular fragility reflects the clinically recognized condition known as repeat concussion and second impact syndrome, where a second trauma to the head occurring prior to the recovery from the first injury results in an exaggerated biological reaction [64,65]. It appears that there is a similar limited capacity for restoration from sub-failure damage that contributes to decreased cellular resistance to mechanical strain following repeat shock wave application in our study. These results point to the directionally dependent susceptibility of the cytoskeletal network to mechanical disruption, which could be due to an anisotropic mechanotransductive response [66]. Therefore, such reports are essential for understanding the mechanopathology of bTBI and guiding the design of therapeutic interventions or protective strategies.

Finally, cell viability remained high (98.5%) for all the shock wave exposure conditions. These are consistent with findings from other groups applying a similar or high-strength shockwave blast using animal or organoid models [67,68]. It should be noted that the viability analyses performed in the current study were unable to account for any cells that may have been lost due to detachment because of the shockwave blast exposure. Additionally, cells occasionally detached during transport between research buildings where the different elements of the experiments were conducted, though this loss of cells would likely be equivalent between the different experimental and control conditions. Statistical analysis showed no difference between the average number of cells remaining adhered to the substrates between the experimental and control conditions at each time point tested for all but one condition. Following single blast exposure from top-to-bottom, the average number of cells was statistically higher for the experimental condition compared to the control condition at 13 DIV (79 cells vs 55 cells, respectively). Nevertheless, the cell viability values obtained for this study represent the viability percentages for the cells that remained adhered to the substrates post-shockwave exposure. Our immunocytochemistry results showed that double shockwave exposure from ‘top-to-bottom’ caused significant changes in cell differentiation. Specifically, significant decreases in the expression of the immature neuron and oligodendrocyte markers, TuJ1 and RIP, respectively, following double shockwave exposure became evident. These findings are well aligned with other studies that found that more severe injuries can exacerbate neuronal trauma, leading to immediate damage that varies with injury severity and cellular properties [18,69]. Previous findings from our group showed that the percentage of maturing neurons and oligodendrocytes present in AHPC neurosphere cultures decreased in a near stepwise fashion with increasing shockwave strength [70]. The current results appear to agree with these findings emphasizing the fact that while the shockwave strength remained constant, increasing the number of blasts may have a similar negative effect on AHPC cell differentiation. Interestingly, when the shockwave blast was applied to the culture dish for ‘bottom-to-top’ double shockwave exposures, there was a slight increase in the percentage of TuJ1 expressing cells. Other *in-vivo* studies have shown that increased neurogenesis occurs around the injured brain regions [27,71,72] in both rat and human brains. Therefore, an explanation for the increase in the amount of TuJ1 present in these cultures supports that cells committed to a neuronal lineage entering an early stage of neuronal development following double shockwave exposure (from ‘bottom-to-top’ direction) to compensate for the loss of neurons when the overpressure threshold is violated in the live cells due to increased an increased amount of impact was coming from below the plate as opposed to above the dish [73]. Therefore, our results showcase how neurons and glial cells maintain their morphology and connectivity in post-injury conditions and further exacerbate symptoms and impede recovery. The above studies show that the cellular and extracellular adaptations to mechanical load are with regard to the responsiveness of the brain to injury, modifying further its vulnerability to additional trauma, and pathophysiological progression after TBI. These findings underline that the responses of cells to mechanical trauma are dynamic and time-dependent and illustrate the importance of integrating multiple approaches of analysis for capturing immediate and long-term effects of shockwave exposure.

We did not examine long-term differentiation due to the limited scope of the study, as we were focused on the more immediate impact of the shockwave blast exposure, as well as the lack of robust differences between the control and shockwave conditions at the later time points analyzed (12 or 10 DIV of total culture time). We also emphasize that our findings should be interpreted with consideration of the complex shockwave dynamics occurring at the air-liquid interface. Because the acoustic impedance of water (or culture medium) is roughly 3500x of air [74], the liquid-air interface behaves similarly to a rigid boundary during shockwave transmission. Prior studies showed that when a shock wave strikes an air-water interface, it is predominantly reflected with phase inversion [75]. It results in the conversion of the initial compressive pulse into a tensile (negative-pressure) wave within the liquid phase. Such tensile stresses can substantially increase the likelihood of cavitation. In practice, we expect the incident blast pulse to be strongly attenuated and inverted upon entering the medium. Importantly, we did not measure the shock waveform inside the liquid, so the exact transmitted pressure history at the cells is unknown. Future investigations incorporating in situ pressure measurements and computational modeling of shockwave transmission through the air-liquid interface will be essential for accurately characterizing the mechanical environment at the cellular level.

## Conclusion

In summary, shockwaves were generated to simulate the bTBI and applied in different magnitudes, durations, and directions to AHPCs and investigated the structural, nanomechanical, and viscoelastic properties as well as cell survival, differentiation, and proliferation changes using AFM and ICC assays, respectively. Nanomechanical property analyzes showed that cells became softer and more deformable with increasing magnitude of shockwaves, especially ‘bottom-to-top’ shockwave set-up due to the disruptive effect on the actin cytoskeletal network. Cells also showed lower viscosity and shorter relaxation times, managing to regain their original shape faster after being exposed to a higher magnitude of shockwave. Although such structural changes were observed, PI staining results showed that cell viability remained consistently high throughout all conditions. However, it has to be noted that these viability values only correspond to the surviving cells that remained adhered to the substrates post-exposure, since the detached cells were not accounted for. Shockwave exposure also demonstrated limited effects on AHPC cell proliferation and differentiation under most conditions; however, there was a significant decline in TuJ1 and RIP expression after double shockwave exposure from ‘top-to-bottom’ direction, which indicates a reduced population of immature neurons and oligodendrocytes, respectively. Conversely, double shockwave exposure from ‘bottom-to-top’ direction resulted in a slight increase in TuJ1 expression indicating more cells committed to a neuronal lineage as a result of compensatory response after injury from higher intensity blast, crossing the overpressure magnitude threshold inside cells. Our results indicated that acoustic impedance mismatches at media interfaces, which control directional variations in shockwave delivery, significantly alter energy transmission and the resulting mechanical disruption of cells. These findings underline that the responses of cells to mechanical trauma are dynamic and time-dependent and illustrate the importance of integrating multiple approaches of analysis for capturing immediate and long-term effects of shockwave exposure. This study is the first of its kind to explore cellular differentiation and cellular nanomechanics simultaneously, immediately following bTBI that can be used as novel biomechanical biomarkers for early diagnosis, prognosis and mechano-responsive regenerative therapies. The biomarkers used here therefore represent early, mechanosensitive indicators of cellular stress and injury rather than downstream biochemical signaling events.

## Supporting information

S1 Fig Experimental timelines for the top-to-bottom and bottom-to-top single and double shockwave exposure experiments. A: Illustrates the culture and experimental timeline for analyses for the top-to-bottom single shockwave exposure samples. B: Illustrates the culture and experimental timeline for analyses of the top-to-bottom double shockwave blast exposure samples. C: Illustrates the culture and experimental timeline for analyses for the bottom-to-top single and double shockwave blast exposure samples.
(PDF)

S1 Table Primary antibodies used during ICC for marking expression of cell proliferation and differentiation. (PDF)

S2 Fig Comparison of viability of AHPCs at 5 DIV following single shockwave exposure from top-to-bottom. A-B: Representative fluorescence images of AHPC viability at 5 DIV following single shockwave exposure. C: At 5 DIV there was no significant difference between the culture conditions (99.85 ± 0.1% vs 99.68 ± 0.1% for control and shock conditions, respectively). Control condition: N = 2 independent experiments; 20 image fields were quantified for this condition. Shock condition: N = 3 independent experiments; 30 image fields were quantified for this condition. **Comparison of Viability of AHPC at 5 DIV Following Double Shockwave Exposure from Top-to-Bottom**. D-E: Representative fluorescence images of AHPC viability after 5 DIV following double shockwave exposure. F: At 5 DIV there was no significant difference between the culture conditions (99.89 ± 0.04% vs 99.03 ± 0.2% for control and shock conditions, respectively). G-H: Propidium iodide reagent controls. Two representative fluorescent images: virtually all cells were stained with the PI following exposure to 70% ethanol. N = 3 independent experiments; 30 image fields were quantified for each condition. Bars represent the mean percentage of viable cells, and the error bars represent the standard error of the mean (±SEM).
(PDF)

S3 Fig Comparison of viability of AHPCs at 13 DIV following single shockwave exposure from top-to-bottom. A-B: Representative fluorescence images of AHPC viability on coverslips after 13 DIV following single shockwave exposure. C: There was no significant difference between the culture conditions (99.88 ± 0.1% vs 99.81 ± 0.1% for control and shock conditions, respectively. N = 2 independent experiments; 20 image fields were quantified for each condition. **Comparison of Viability of AHPCs at 12 DIV Following Double Shockwave Exposure from Top-to-Bottom**. D-E: Representative fluorescence images of AHPC viability on coverslips after 12 DIV following single shockwave exposure. F: There was no significant difference between the culture conditions (99.17 ± 0.3% vs 98.8 ± 0.4% for control and shock conditions, respectively). N = 3 independent experiments; 30 image fields were quantified for each condition. Bars represent the mean percentage of viable cells, and the error bars represent the standard error of the mean (±SEM).
(PDF)

S4 Fig Comparison of viability of AHPCs at 5 DIV following single or double shockwave exposure from top-to-bottom. A: Quantitative analysis of AHPCs at 5 DIV following single or double shockwave exposure from Top-to-Bottom. There was a significant decrease in cell viability following double shockwave exposure compared to single shockwave exposure. There was no significant difference between the control culture conditions. N = 3 independent experiments; 30 image fields were quantified for each condition. **Comparison of Viability of AHPCs at 12–13 DIV Post-Expansion Following Single or Double Shockwave Exposure from Top-to-Bottom**. B: Quantitative analysis of AHPCs on coverslips at 12–13 DIV following single or double shockwave exposure from above. There was a significant decrease in cell viability following double shockwave exposure compared to single shockwave exposure for both the control and exposed culture conditions. N = 3 independent experiments; 30 image fields were quantified for each condition. **Comparison of Differentiation and Proliferation of AHPCs at 12–13 DIV Post-Expansion Following Single or Double Shockwave Exposure from Top-to-Bottom**. C: Quantitative analysis of AHPCs on coverslips at 12–13 DIV post-expansion following single or double shockwave exposure from above. There was a significant decrease in the expression of TuJ1 following double shockwave exposure compared to single shockwave exposure. There was no significant difference between exposure conditions for the other antibodies tested. Single shock condition: N = 2 independent experiments; 20 image fields were quantified for this condition. Double shock condition: N = 3 independent experiments; 30 image fields were quantified for this condition. Bars represent the mean percentage of viable cells, and the error bars represent the standard error of the mean (±SEM).
(PDF)

S5 Fig Comparison of viability of AHPCs at 4 DIv following single shockwave exposure from bottom-to-top. A-B: Representative fluorescence images of AHPC viability at 4 DIV following single shockwave exposure. C: At 4 DIV, there was no significant difference between the culture conditions (99.45 ± 0.2% vs 99.6 ± 0.2% for control and shock conditions, respectively). N = 3 independent experiments, 30 image fields were quantified for each condition. **Comparison of Viability of AHPC at 4 DIV Following Double Shockwave Exposure from Bottom-to-Top**. D-E: Representative fluorescence images of AHPC viability at 4 DIV following double shockwave exposure. F: At 4 DIV, there was no significant difference between the culture conditions (98.55 ± 0.5% vs 98.73 ± 0.5% for control and shock conditions, respectively). N = 3 independent experiments, 30 image fields were quantified for each condition. Bars represent the mean percentage of viable cells, and the error bars represent the standard error of the mean (±SEM).
(PDF)

S6 Fig Comparison of viability of AHPCs at 10 DIV following single shockwave exposure from bottom-to-top. **A-B**: Representative fluorescence images of AHPC viability on coverslips after 10 DIV following single shockwave exposure. C: There was no significant difference between the culture conditions (99.21 ± 0.3% vs 99.45 ± 0.2% for control and shock conditions, respectively). N = 3 independent experiments; 30 image fields were quantified for each condition. **Comparison of Viability of AHPCs at 10 DIV Following Double Shockwave Exposure from Bottom-to-Top. D-E**: Representative fluorescence images of AHPC viability on coverslips after 10 DIV following single shockwave exposure. F: There was no significant difference between the culture conditions (99.26 ± 0.3% vs 99.12 ± 0.3% for control and shock conditions, respectively). N = 3 independent experiments; 30 image fields were quantified for each condition. Bars represent the mean percentage of viable cells, and the error bars represent the standard error of the mean (±SEM).
(PDF)

S7 Fig Comparison of viability of AHPCs at 4 DIV following single or double shockwave exposure from bottom-to-top. A: Quantitative analysis of AHPCs at 4 DIV following single or double shockwave exposure from Bottom-to-Top. There was no significant difference between the control or shockwave culture conditions for either exposure condition. **Comparison of Viability of AHPCs at 10 DIV Following Single or Double Shockwave Exposure from Bottom-to-Top**. B: Quantitative analysis of AHPCs on coverslips at 10 DIV following single or double shockwave exposure from Bottom-to-Top. There was no significant difference between the control or shockwave culture conditions for either exposure condition. **Comparison of Differentiation and Proliferation of AHPCs at 10 DIV Following Single or Double Shockwave Exposure from Bottom-to-Top.** C: Quantitative analysis of AHPCs on coverslips at 10 DIV following single or double shockwave exposure from Bottom-to-Top. There was a significant increase in the expression of TuJ1 following double shockwave exposure compared to single shockwave exposure. There was no significant difference between exposure conditions for the other antibodies tested. N = 3 independent experiments; 30 image fields were quantified for each condition in plots. Bars represent the mean percentage of viable cells, and the error bars represent the standard error of the mean (±SEM).
(PDF)

S8 Fig Phase contrast images of AHPCs under controlled study (A) and the cells being exposed to shockwave (B-E), both kept in the incubator for 3 hours after the shockwave exposure, showed the restoration process. By observing these images, cells were gradually recovering from their blebbing stage to regular cell morphology.
(PDF)
