# Supplementary material for: The effects of blast-induced traumatic brain injury on brain cellular mechanics and differentiation
Source: PLoS One. 2026 Aug 13;21(8):e0355739. doi: 10.1371/journal.pone.0355739 (PMC13472447; doi:10.1371/journal.pone.0355739)
Supplement: Supplementary file 1 [file pone.0355739.s001.pdf]

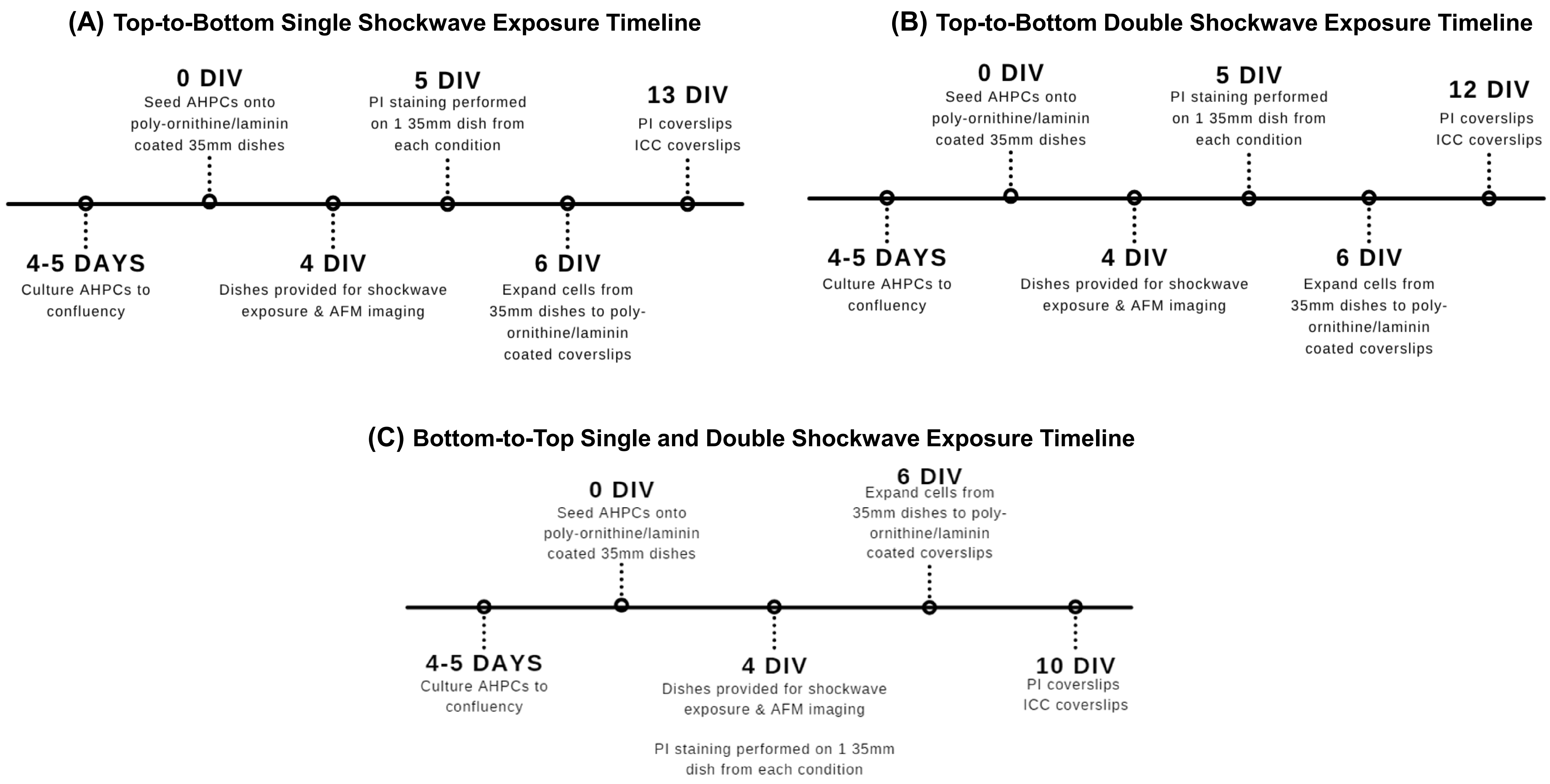
(A) Top-to-Bottom Single Shockwave Exposure Timeline
0 DIV
Seed AHPCs onto poly-ornithine/laminin coated 35mm dishes
5 DIV
PI staining performed on 1 35mm dish from each condition
13 DIV
PI coverslips ICC coverslips
4-5 DAYS
Culture AHPCs to confluency
4 DIV
Dishes provided for shockwave exposure & AFM imaging
6 DIV
Expand cells from 35mm dishes to poly-ornithine/laminin coated coverslips
(B) Top-to-Bottom Double Shockwave Exposure Timeline
0 DIV
Seed AHPCs onto poly-ornithine/laminin coated 35mm dishes
5 DIV
PI staining performed on 1 35mm dish from each condition
12 DIV
PI coverslips ICC coverslips
4-5 DAYS
Culture AHPCs to confluency
4 DIV
Dishes provided for shockwave exposure & AFM imaging
6 DIV
Expand cells from 35mm dishes to poly-ornithine/laminin coated coverslips
(C) Bottom-to-Top Single and Double Shockwave Exposure Timeline
0 DIV
Seed AHPCs onto poly-ornithine/laminin coated 35mm dishes
6 DIV
Expand cells from 35mm dishes to poly-ornithine/laminin coated coverslips
4-5 DAYS
Culture AHPCs to confluency
4 DIV
Dishes provided for shockwave exposure & AFM imaging
PI staining performed on 1 35mm dish from each condition
10 DIV
PI coverslips ICC coverslips
