# Supplementary material for: The effects of blast-induced traumatic brain injury on brain cellular mechanics and differentiation
Source: PLoS One. 2026 Aug 13;21(8):e0355739. doi: 10.1371/journal.pone.0355739 (PMC13472447; doi:10.1371/journal.pone.0355739)
Supplement: Supplementary file 2 [file pone.0355739.s002.pdf]

Table S1. Primary antibodies used during ICC for marking expression of cell proliferation and differentiation.

Antibody	Cell Marker
Ki67	Cell Proliferation
Tuj1	Immature Neurons
MAP2ab	Maturing Neurons
RIP	Oligodendrocytes
GFAP	Astrocytes
