# Supplementary material for: The effects of blast-induced traumatic brain injury on brain cellular mechanics and differentiation
Source: PLoS One. 2026 Aug 13;21(8):e0355739. doi: 10.1371/journal.pone.0355739 (PMC13472447; doi:10.1371/journal.pone.0355739)
Supplement: Supplementary file 5 [file pone.0355739.s005.pdf]

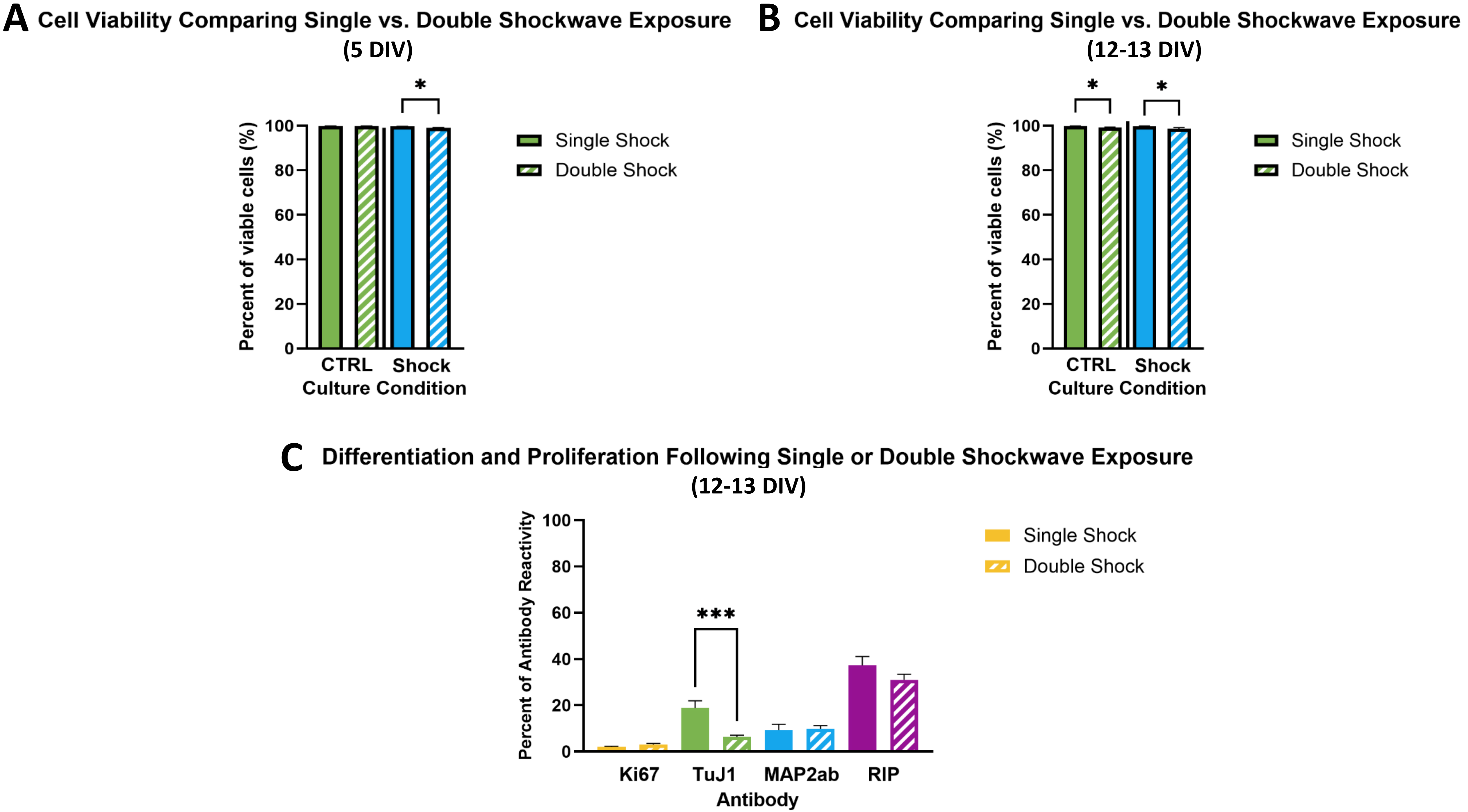
A
Cell Viability Comparing Single vs. Double Shockwave Exposure
(5 DIV)
*
Percent of viable cells (%)
100
80
60
40
20
0
CTRL
Shock
Culture Condition
Single Shock
Double Shock
B
Cell Viability Comparing Single vs. Double Shockwave Exposure
(12-13 DIV)
*
*
Percent of viable cells (%)
100
80
60
40
20
0
CTRL
Shock
Culture Condition
Single Shock
Double Shock
C
Differentiation and Proliferation Following Single or Double Shockwave Exposure
(12-13 DIV)
***
Percent of Antibody Reactivity
100
80
60
40
20
0
Ki67
TuJ1
MAP2ab
RIP
Antibody
Single Shock
Double Shock
