# Supplementary material for: The effects of blast-induced traumatic brain injury on brain cellular mechanics and differentiation
Source: PLoS One. 2026 Aug 13;21(8):e0355739. doi: 10.1371/journal.pone.0355739 (PMC13472447; doi:10.1371/journal.pone.0355739)
Supplement: Supplementary file 6 [file pone.0355739.s006.pdf]

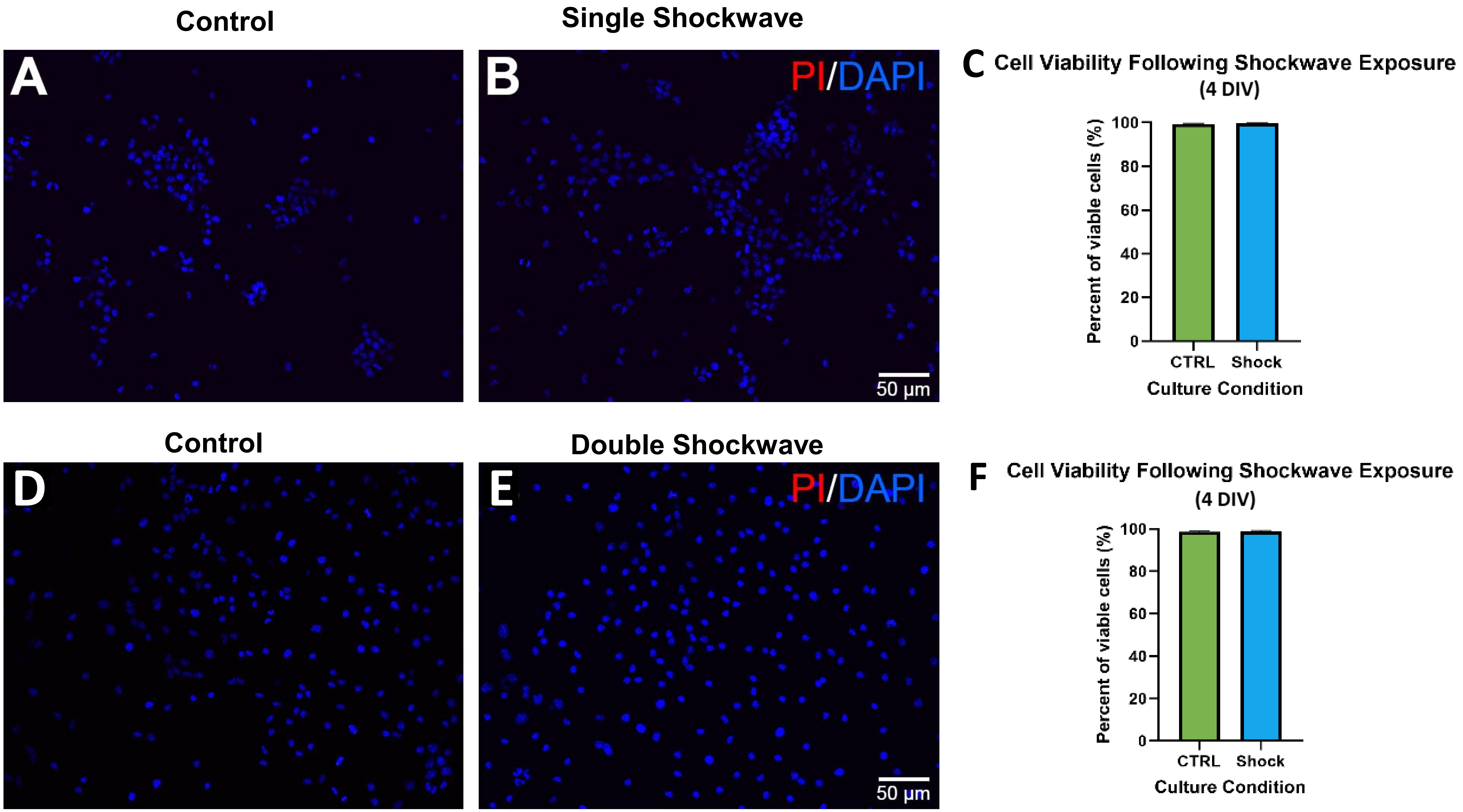
Control
Single Shockwave
A
B
PI/DAPI
50 µm
C
Cell Viability Following Shockwave Exposure
(4 DIV)
Percent of viable cells (%)
100
80
60
40
20
0
CTRL
Shock
Culture Condition
Control
Double Shockwave
D
E
PI/DAPI
50 µm
F
Cell Viability Following Shockwave Exposure
(4 DIV)
Percent of viable cells (%)
100
80
60
40
20
0
CTRL
Shock
Culture Condition
