# Supplementary material for: The effects of blast-induced traumatic brain injury on brain cellular mechanics and differentiation
Source: PLoS One. 2026 Aug 13;21(8):e0355739. doi: 10.1371/journal.pone.0355739 (PMC13472447; doi:10.1371/journal.pone.0355739)
Supplement: Supplementary file 8 [file pone.0355739.s008.pdf]

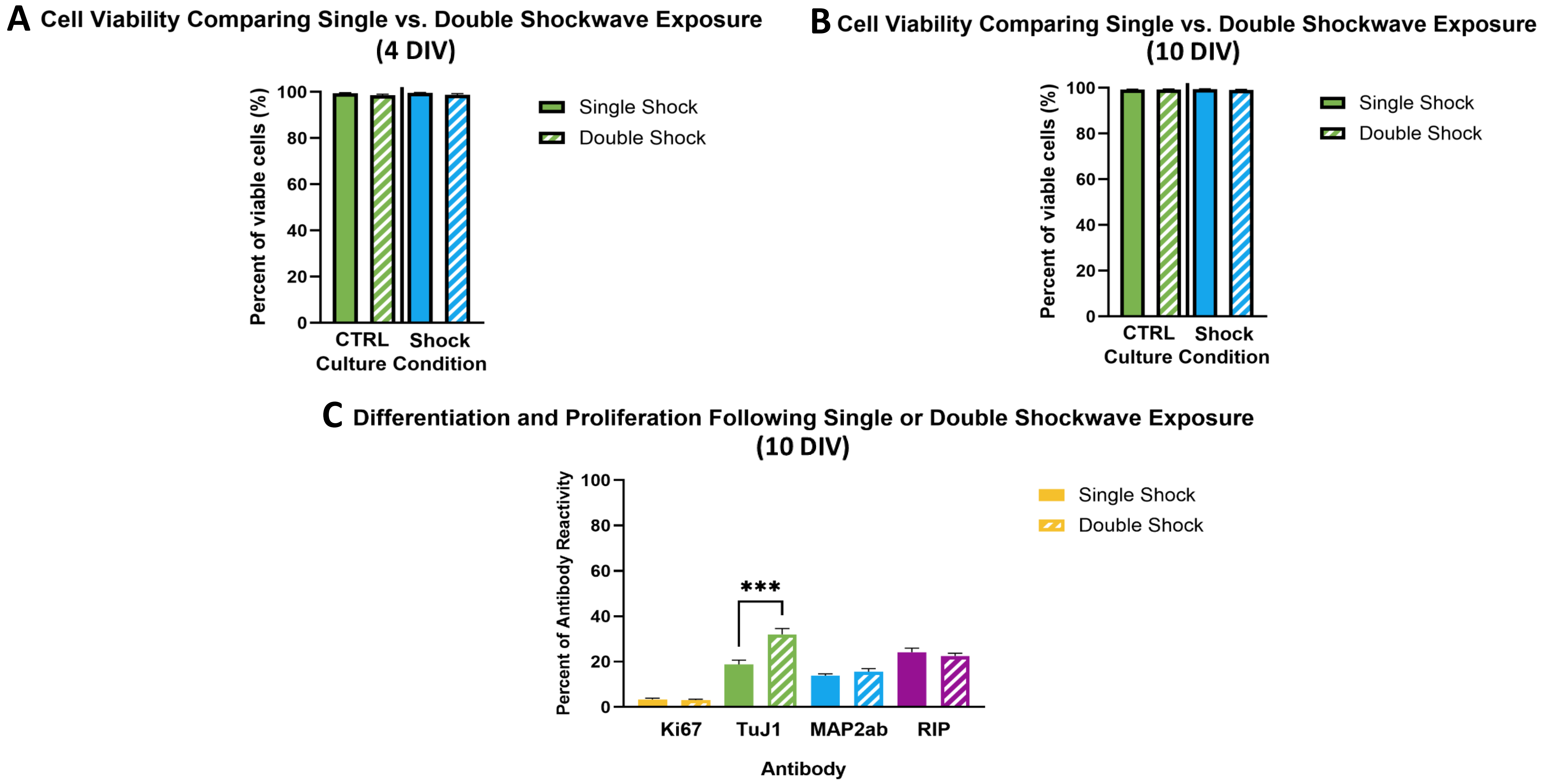
A Cell Viability Comparing Single vs. Double Shockwave Exposure (4 DIV)
Percent of viable cells (%)
100
80
60
40
20
0
CTRL
Shock
Culture Condition
Single Shock
Double Shock
B Cell Viability Comparing Single vs. Double Shockwave Exposure (10 DIV)
Percent of viable cells (%)
100
80
60
40
20
0
CTRL
Shock
Culture Condition
Single Shock
Double Shock
C Differentiation and Proliferation Following Single or Double Shockwave Exposure (10 DIV)
Percent of Antibody Reactivity
100
80
60
40
20
0
***
Ki67
TuJ1
MAP2ab
RIP
Antibody
Single Shock
Double Shock
