# Supplementary material for: The effects of blast-induced traumatic brain injury on brain cellular mechanics and differentiation
Source: PLoS One. 2026 Aug 13;21(8):e0355739. doi: 10.1371/journal.pone.0355739 (PMC13472447; doi:10.1371/journal.pone.0355739)
Supplement: Supplementary file 9 [file pone.0355739.s009.pdf]

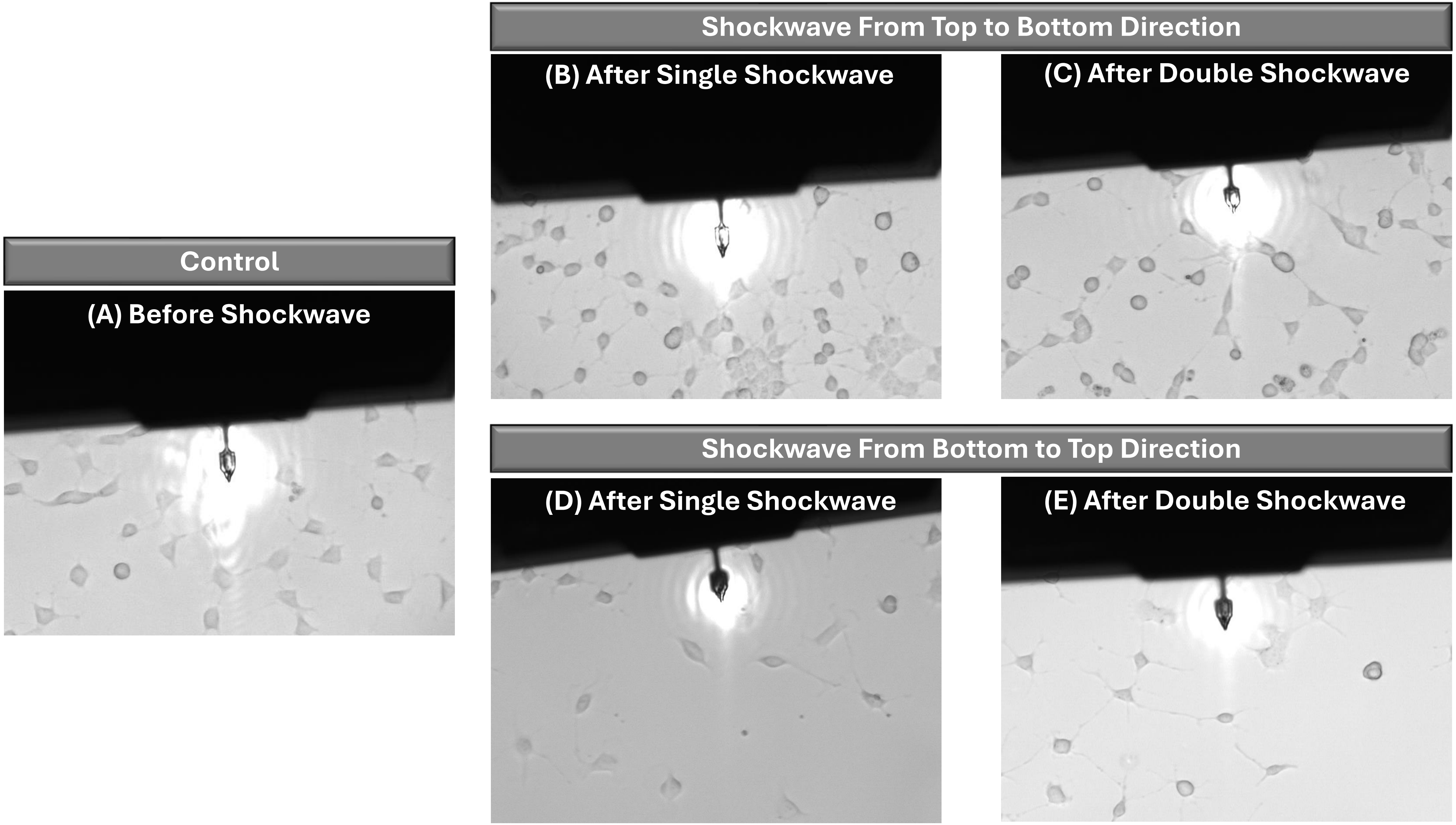
Control
(A) Before Shockwave
Shockwave From Top to Bottom Direction
(B) After Single Shockwave
(C) After Double Shockwave
Shockwave From Bottom to Top Direction
(D) After Single Shockwave
(E) After Double Shockwave
