# Supplementary material for: The effects of blast-induced traumatic brain injury on brain cellular mechanics and differentiation
Source: PLoS One. 2026 Aug 13;21(8):e0355739. doi: 10.1371/journal.pone.0355739 (PMC13472447; doi:10.1371/journal.pone.0355739)
Supplement: Supplementary file 10 [file pone.0355739.s011.pdf]

Response to Reviewers

The effects of blast-induced traumatic brain injury on brain cellular mechanics and differentiation

We extend our appreciation to the editor for giving us the opportunity to revise our manuscript, taking into consideration the constructive feedback provided by the reviewer. We hope that with these changes (highlighted in the paper with red color), the paper can be accepted without any further revisions. We have addressed the individual concerns of the reviewers in detail below. We look forward to the comments on our revised version.

Reviewer #1:

We thank the reviewer for the feedback on our manuscript.

1. **Methods:** For basic experimental studies, detailed methodology is essential. The manuscript should provide a clear and comprehensive description of the experimental procedures.

   Response: We thank the reviewer for highlighting the need for additional methodological clarity. In response, we have expanded the Cell Culture subsection of the Materials and Methods. The revised text now includes:

   - More explicit descriptions of AHPC culture conditions.
   - Detailed seeding densities, timing, and procedures.
   - Clarified timelines for pre-shockwave culture, post-shockwave recovery, expansion onto coverslips, and differentiation protocols.
   - Precise specifications of coating procedures for poly-L-ornithine/laminin substrates.

   These revisions are intended to ensure full reproducibility and transparency of the experimental workflow. All added information appears in the **Materials and Methods** (page: 4-5, line: 108-157) and the revised paragraph is added below:

   Cell Culture:

   "Adult rat hippocampal progenitor cells (AHPCs) were gifted by Dr. F.H. Gage, Salk Institute, La Jolla, CA. The AHPCs were isolated from the hippocampus of adult Fisher 344 rats with prior approval from the Animal Care and Use Committee of the Salk Institute. AHPCs were cultured in a T25 tissue culture flask (Thermo Fisher Scientific) coated with poly-L-ornithine (10 µg/mL; Sigma Aldrich) overnight at room temperature, rinsed with an excess volume of phosphate buffered saline (PBS) the following day, and incubated with a laminin (10 µg/mL; Cultrex by Trevigen) solution made in PBS. AHPCs were then incubated overnight at 37℃ in 5% CO_2 incubator. The following day the laminin solution was removed, rinsed with PBS and stored frozen until use. Cells were cultured in maintenance medium (MM) composed of Dulbecco's modified Eagle's medium/Ham's F-12 (DMEM/F-12, 1:1; Gibco by Thermo Fisher Scientific) supplemented with 2.5 mM GlutaMAX (Thermo Fisher Scientific), 1 x N2 supplement (Gibco by Thermo Fisher Scientific), 1 x Penicillin/streptomycin (Gibco by Thermo Fisher Scientific), and 20 ng/mL basic fibroblast growth factor (bFGF; Gibco by Thermo Fisher Scientific). The cells were incubated at 37℃ in a 5% CO2 atmosphere. AHPCs in the flask were fed with MM every 2 days by performing half media changes. They were monitored daily to ensure they were contamination-free and harvested when the cell confluency reached 80%. The multipotential nature of the AHPCs was verified by immunocytochemistry.

   Top-to-Bottom Shockwave Exposure Experiments:

   Cells were initially seeded into poly-L-ornithine/laminin (POL) coated 35 mm petri dishes (Falcon) at a density of 40,000 cells/dish. Prior to seeding, cell counts were performed using Trypan Blue (Gibco) and a hemocytometer. Dishes were maintained in MM for 6 days in vitro (DIV) with standard half media changes. At 4 DIV, a subset of the dishes was exposed to shockwave blast injury. The cells within the dishes were cultured for an additional 48 hours at which time the cells from the dishes were harvested by adding warmed 0.05% Trypsin EDTA solution (Corning) to the dishes and placing the dishes in an incubator for 2 minutes. Cells were then resuspended with MM and transferred to a 15 mL centrifuge tube and centrifuged for 6 minutes at 300 RCF. After cell pellet was formed, supernatant was removed and cells were resuspended with MM. The harvested cells were then expanded onto POL-coated coverslips at a density of 3,000 cells/coverslip. Half of the coverslips were seeded with non-exposed cells while the remaining half were seeded with cells exposed to the shockwave blast. Coverslips were cultured in MM for the first 24 hours after which time the media was switched to differentiation medium (DM; MM without the

bFGF), and the cells were maintained in DM until the end of the culture period. For the single shockwave blast exposure experiments the coverslips were maintained until 13 DIV, and for the double shockwave blast exposure the coverslips were maintained until 12 DIV (S1 Fig).

Bottom-to-Top Shockwave Exposure Experiments:

Cells were initially seeded into POL-coated 35 mm petri dishes at a density of 20,000 cells/dish. Dishes were maintained in MM for 6 DIV with standard half media changes. At 4 DIV, a subset of the dishes was exposed to shockwave blast injury. The cells within the dishes were cultured for an additional 48 hours at which time the cells from the dishes were harvested and expanded onto POL-coated coverslips at a density of 2,000 cells/coverslip as previously described. Half of the coverslips were seeded with non-exposed cells while the remaining half were seeded with cells exposed to the shockwave blast. Coverslips were cultured in MM for the first 24 hours and the media was switched to DM after that. The cells were maintained in DM until the end of the culture period. For both the single and double shockwave exposure experiments the coverslips were maintained until 10 DIV (S1 Fig)."

Additionally, in the original paper, the methodology for the shock tube was placed in the Results section, in the subsection entitled "Shockwave Generation and Propagation". The subsection "Shockwave Generation and Propagation" has now been moved to the section called **"Materials and methods"** on pages 3-4 (line: 74-106) for clarity. Also, Figure 1B (Page: 2) has been updated with the computer-aided draft (CAD) rendering of the shock tube to enhance clarity and the caption for Figure 1B has been updated accordingly as follows:

"(B) Schematic of a shock tube device employed to produce controlled shockwaves. It has a driven section (blue) holding cell cultures (light blue) and a driver section (red), divided by a diaphragm. When activated by pressurizing the driver section with compressed air, the diaphragm bursts and creates the shockwave that propagates through the driven section towards the cells. The cells within the substrates are clamped to the end of the driven section of the shock tube to facilitate shockwave exposure."

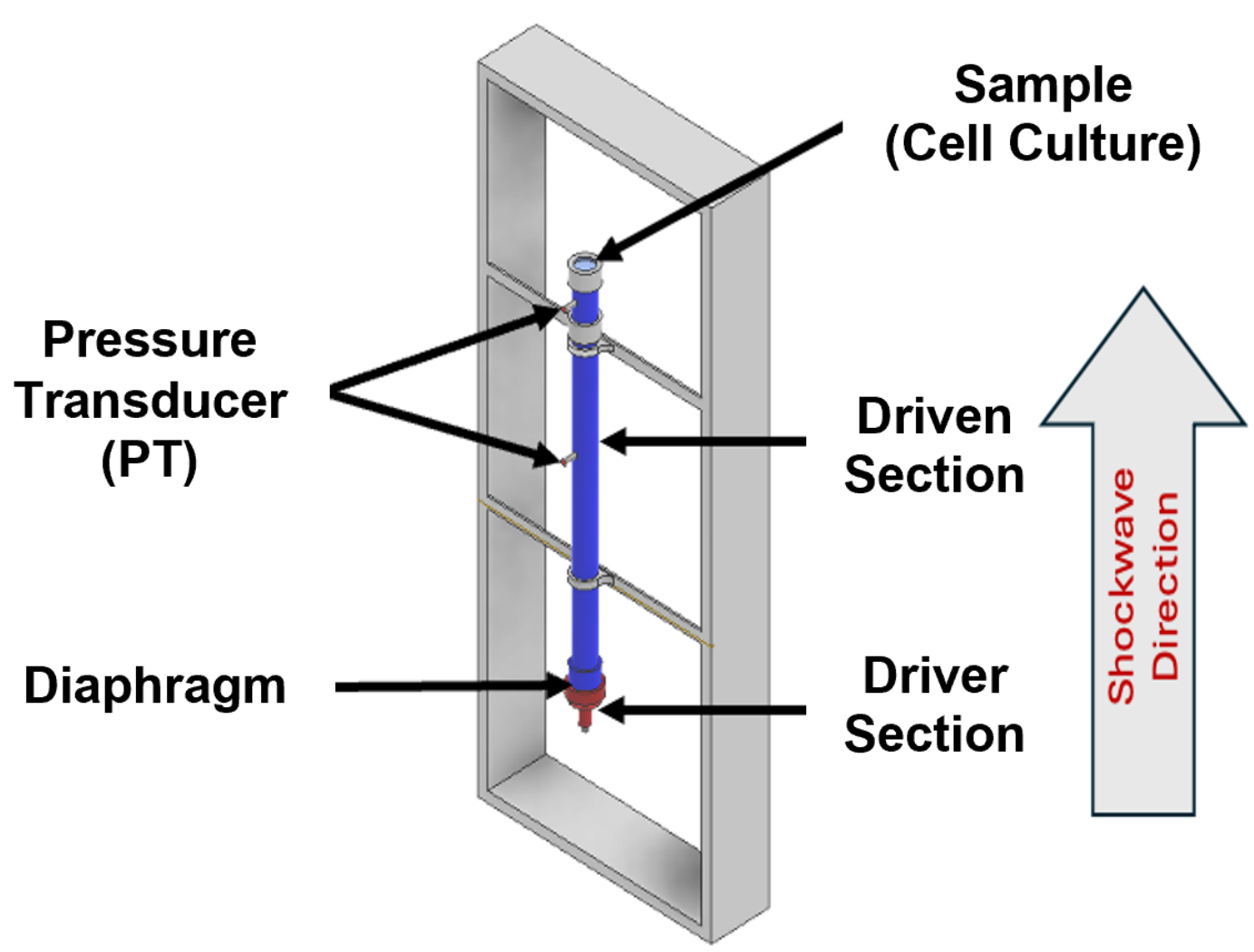

Figure 1: "(B) Schematic of a shock tube device employed to produce controlled shockwaves. It has a driven section (blue) holding cell cultures (light blue) and a driver section (red), divided by a diaphragm. When activated by pressurizing the driver section with compressed air, the diaphragm bursts and creates the shockwave that propagates through the driven section towards the cells. The cells within the substrates are clamped to the end of the driven section of the shock tube to facilitate shockwave exposure."

Furthermore, additional methodological details have been included in the ***Atomic Force Microscope (AFM) Setup*** on pages 5-6 (line: 165-183) subsection of the Materials and Methods to more improve clarity.

"Cells were cultured for 72 h under standard growth conditions to reach the desired confluency and were then exposed to single or double blast-induced shockwaves at defined pressure levels using the shock tube apparatus, as detailed in the shockwave exposure protocol. Immediately after shockwave exposure, the dish with AHPCs was placed on the AFM stage (connected to the sample heater) in a vibration isolation chamber to maintain a controlled temperature environment during measurements. Control cells were also retrieved from the incubator alongside treated cells to maintain consistency for comparison. Pre-calibrated PFQNM-LC-CAL-A probes,

specifically designed for live cell applications, were employed for single-cell measurements. These probes, with a 70 nm tip radius and a nominal spring constant ranging from 0.074 N/m to 0.101 N/m, offered the necessary sensitivity and flexibility for probing soft biological materials without inducing excessive deformation. A controlled force of 300 pN was applied during each indentation, ensuring gentle yet consistent contact with the cell membrane and minimizing structural disruption. The AFM scan rate and scan size were set at 0.242 Hz and ranging from 17 μm to 50 μm, respectively, with 256x256 pixel size, achieving a balance between spatial resolution and throughput. This allowed for accurate nanomechanical mapping of each cell's surface topography, capturing critical mechanical properties."

2. **Blast model orientation:** The rationale and significance of the "top-to-bottom" versus "bottom-to-top" exposure need to be explicitly stated and discussed in more detail.

   Response: We thank the reviewer for this constructive comment regarding the need to clarify the rationale and significance of the 'top-to-bottom' versus 'bottom-to-top' exposure. In response, we have explicitly addressed this aspect by adding the following to the **Discussion** section, where the experimental rationale and its biological and biomechanical significance are clearly explained on pages 13-14 (line: 485-509) of the revised manuscript.
   "Blast-induced trauma is strongly influenced by the presence of intermediate protective barriers between the source of the blast and the target cell, which is the underlying nervous tissue. In reality, particularly in a combat situation, the possibility that the pressure wave from the explosion will be in direct contact with nervous tissue is extremely low, as it travels along various layers of protection such as helmets, padding systems, skull bone, cerebrospinal fluid, and soft tissue. Each of these materials has unique mechanical impedance properties, which can either reflect, refract, attenuate, or focus stress waves incident upon it, depending upon their intensities, before they reach neural cells. It has been shown that the directionality of the blast wave compared to anatomical geometry makes a significant contribution to the intracranial pressure distribution, shear stress localization, and tissue deformation patterns (Moss et al., 2009; Taylor and Ford, 2009; Ganpule et al., 2012). Thus, overhead explosions or airborne detonations may undergo a different pattern of reflection and redistribution by helmet shells and skull curvature compared to the ground-based improvised explosion that can transmit energy more directly through the cervical and basal cranial structures. Also, while protective equipment is intended to lessen the severity of the maximum injuries, the presence of multiple layers does not act to reduce the impact of the blast in a consistent manner. Instead, depending on the severity of the blast and the direction, protective equipment can produce inadvertent stress concentrations, as well as the effect of wave focusing, within the skull. Computer simulations and other research have indicated the use of helmets can alter the shape of the blast wave, which can decrease the maximum pressure while increasing the impulse or internal heterogeneity of stress (Moss et al., 2009; Ganpule et al., 2012).
   In this regard, our comprehensive investigation simulated blast waves in a controlled laboratory setting and examined the biomechanical and developmental responses of AHPCs after exposure to mechanical shock waves of different magnitudes and directions."

   38. Moss WC, King MJ, Blackman EG. Skull flexure from blast waves: a mechanism for brain injury with implications for helmet design. Phys Rev Lett. 2009;103(10):108702.
   39. Taylor PA, Ford CC. Simulation of blast-induced early-time intracranial wave physics leading to traumatic brain injury. J Biomech Eng. 2009;131(6):061007.
   40. Ganpule S, Alai A, Plougonven E, Chandra N. Mechanics of blast loading on the head models in the study of traumatic brain injury using experimental and computational approaches. Biomech Model Mechanobiol. 2013;12(3):511–531.

3. **Ethical considerations:** If animal-derived cells or models were used, the ethical approval and considerations should be described.

   Response: We thank the reviewer for this comment. In response, we have added the following information to the Cell Culture subsection of the Materials and Methods on page 4 (line: 108-111).
   "Adult rat hippocampal progenitor cells (AHPCs) were gifted by Dr. F.H. Gage, Salk Institute, La Jolla, CA. The AHPCs were isolated from the hippocampus of adult Fisher 344 rats with prior approval from the Animal Care and Use Committee of the Salk Institute."

   Clinical implications: The statements on regenerative medicine and personalized medicine are currently speculative. These should either be removed or supported with appropriate evidence and references.

Response: We would like to thank the reviewer for the suggestion. We have clarified this by adding the following line with reference to the revised manuscript (page: 3, line: 34-42).

Furthermore, a study [20] showed that mechanical forces play a critical role in modulating stem cell behavior by influencing their proliferation and differentiation. It was also found that the mechanical environment and stiffness of CNS tissue or biomaterials can affect the differentiation, adhesion, and migration of stem cells. Biomaterial mechanics matching to CNS tissue is important for regenerative medicine because such mechanical cues strongly modulate cellular behavior and the success of therapeutic strategies [21].These findings highlight the importance of investigating how mechanical forces affect the nanomechanical properties of stem cells following blast trauma that may contribute to advancements in bTBI diagnostics and treatment.

21. Bartlett, R. D., Eleftheriadou, D., Evans, R., Choi, D., & Phillips, J. B. (2020). Mechanical properties of the spinal cord and brain: Comparison with clinical-grade biomaterials for tissue engineering and regenerative medicine. Biomaterials, 258, 120303.

4. **Figures:** A schematic or actual photograph of the blast model setup would greatly help readers understand the experimental system.

Response: We thank the reviewer for their comment. Figure 1B has been updated with the computer-aided draft (CAD) rendering of the shock tube to enhance clarity of the blast model setup. The caption for Figure 1B has also been updated accordingly on page 2.

"(B) Schematic of a shock tube device employed to produce controlled shockwaves. It has a driven section (blue) holding cell cultures (light blue) and a driver section (red), divided by a diaphragm. When activated by pressurizing the driver section with compressed air, the diaphragm bursts and creates the shockwave that propagates through the driven section towards the cells. The cells within the substrates are clamped to the end of the driven section of the shock tube to facilitate shockwave exposure."

Reviewer #2:

We thank the reviewer for the feedback on our manuscript.

1. **Viability Measurement Limitations:** The authors' reliance on propidium iodide (PI) staining fails to account for cells lost to detachment following shockwave exposure. Consequently, reported viability rates ($> 98 - 99\%$) reflect only those cells that remain adherent, not the total population initially present. This undermines all viability statistics, given that cell loss is visible as clumping and empty spaces in the panels.

2. **Unquantified Immediate Cell Loss:** The omission of detached, dead cells from viability counts results in a systematic underestimation of initial trauma-induced damage.

   Response: We agree with the reviewer's comment 1 and 2 that PI-based viability measurements capture only adherent cells and may underestimate initial shockwave-induced detachment, as well as cell loss during transfer of the cells between three different campus locations to conduct the different parts of the experiments (cell culture, shockwave/blast exposure, and, AFM imaging were all conducted at different locations on the university campus). We have added clarifying statements, acknowledging this limitation in both the Results and Discussion sections. (Found in Discussion section, page: 15, line: 574-587)
   "It should be noted that the viability analyses performed in the current study were unable to account for any cells that may have been lost due to detachment because of the shockwave blast exposure. Additionally, cells occasionally detached during transport between research buildings where the different elements of the experiments were conducted, though this loss of cells would likely be equivalent between the different experimental and control conditions. Statistical analysis showed no difference between the average number of cells remaining adhered to the substrates between the experimental and control conditions at each time point tested for all but one condition (data not shown). Following single blast exposure from top-to-bottom, the average number of cells was statistically higher for the experimental condition compared to the control condition at 7 DIV post-expansion (79 cells vs 55 cells, respectively). Nevertheless, the cell viability values obtained for this study represent the viability percentages for the cells that remained adhered to the substrates post-shockwave exposure."

3. **In Vitro Model Context:** Although controlling for variables, the exclusive use of an in vitro model (with AHPCs) diverges significantly from in vivo conditions. Furthermore, the lack of physical integration of substrates and blast setup introduces the possibility of air blast or sound artifacts affecting cells—calling into question the ecological validity of the experimental system.

   Response: We thank the reviewer for their comment. Figure 1B has been updated with the computer-aided draft (CAD) rendering of the shock tube to enhance clarity of how the substrates containing the cells are integrated with the blast model setup. The cells within the substrates are clamped to the end of the driven section of the shock tube to facilitate shockwave exposure.
   Therefore, we also omitted the line "and positioning substrates without being attached to a blast setup, as seen in animal studies" in the revised manuscript to avoid confusion for the reader. Also, the following figure has now been added to Figure 1B (page: 2):

4. **Limited Long-Term Differentiation Data:** While early markers of differentiation are explored at 4 or 6–7 days in vitro (DIV), there is little examination of later-stage differentiation or lineage outcome, limiting conclusions about persistent or functionally relevant changes.

   Response: We would like to thank the reviewer for this comment. In response to the comment, we added the following at the end of the **Discussion** section (page: 16, line: 616-620) of the revised manuscript:
   "We did not examine long term differentiation due to the limited scope of the study as we were focused on the more immediate impact of the shockwave blast exposure as well as the lack of robust differences between the control and shockwave conditions at the later time points analyzed (12 or 10 DIV of total culture time/6-7 DIV post shockwave exposure)."

5. **Inappropriate Use of DAPI:** The manuscript's use of DAPI as a live-cell indicator is highly unconventional, as live cells do not incorporate DAPI. Complete DAPI positivity indicates cell death, further confounding data interpretation in Figures S2 and S3. Moreover, the lack of clear PI staining is suspicious. Employing a proper death control

and established viability assays (e.g., PI and Hoechst 33342 or Calcein/PI combination) would yield more reliable results.

Response: We thank the reviewer for the comment. We have incorporated the following clarification into the **"Propidium iodide (PI) staining"** subsection of the "Materials and Methods" section (Page: 7, Line: 236-240). Also, we have now added images of the PI Reagent Control to Supplementary Figure S2.
"Propidium iodide staining was used to evaluate cell survival. As a positive control for the PI reagent, samples with AHPCs were incubated with 70% ethanol, a condition known to kill most cells, resulting in a high percentage of PI-labeled cells. In all previous studies where we have used PI staining, this reagent control has verified that the PI staining was working properly."
As noted by the reviewer, this PI reagent control is especially valuable when there is minimal PI staining of the cell samples. We also added the following into **"Immunocytochemistry (ICC)"** subsection (Page: 7, Line: 264-267) for clarification.
"The DAPI counterstaining used throughout the results section was performed on paraformaldehyde fixed cells. The DAPI was not used as a live cell marker. The total number of cells in an image field was determined by counting the number of DAPI stained nuclei."

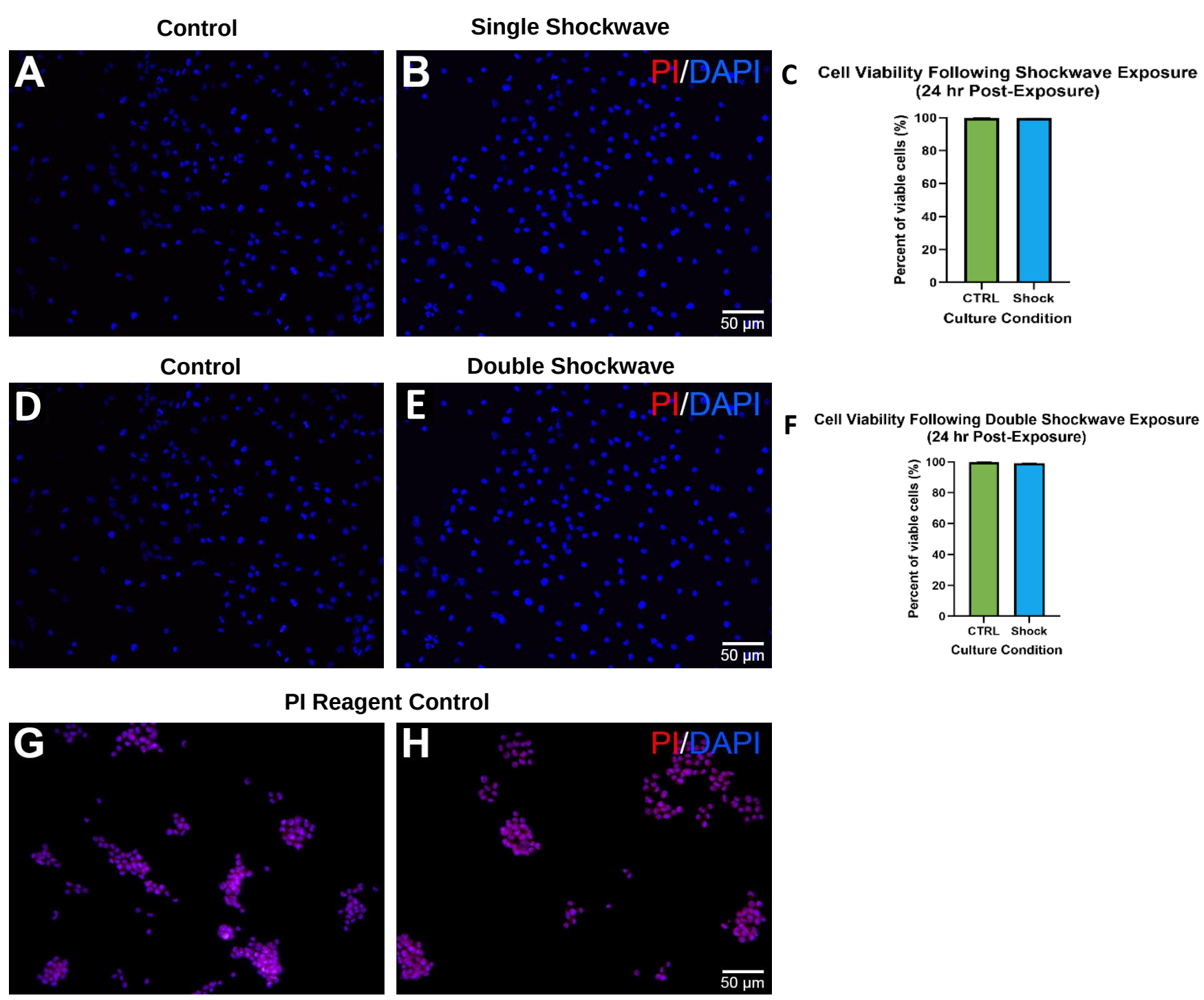

Figure 2: Comparison of Viability of AHPCs at 24 hr. Post-Exposure Following Single Shockwave Exposure from Top-to-Bottom. A-B: Representative fluorescence images of AHPC viability after 24 hr. post-exposure following single shockwave exposure. C: At 24 hrs. post-shockwave there was no significant difference between the culture conditions (99.85±0.1% vs 99.68±0.1% for control and shock conditions, respectively). N=2-3 independent experiments, 20-30 image fields were quantified for each condition. **Comparison of Viability of AHPC at 24 hr. Post-Exposure Following Double Shockwave Exposure from Above.** D-E: Representative fluorescence images of AHPC viability after 24 hr. post-exposure following double shockwave exposure. F: At 24 hours post-shockwave there was no significant difference between the culture conditions (99.85±0.1% vs 99.68±0.1% for control and shock conditions, respectively). G-H: Propidium iodide reagent controls. Two representative fluorescent images: virtually all cells were stained with the PI following exposure to 70% ethanol. N=3 independent experiments, 30 image fields were quantified for each condition. Bars represent the mean percentage of viable cells, and the error bars represent the standard error of the mean (±SEM).

6. **Inconclusive Marker Data:** Most bar graphs depicting up- or downregulation of markers fail to show robust or interpretable effects; double-labeling approaches may be necessary for clarity. For example, the purported decline in viability following double versus single shockwave exposure (Figure S4) is not substantiated by the data, as all groups display nearly 100% viability. Edu as a measure of proliferation would be much more interpretable than the use of Ki67. Tuj1 on its own does not support neurogenesis. Control experiments are missing here.

   Response: We thank the reviewer for these comments. We now added a explaination in the **"Results"** section of using Ki67 (page: 13, line: 473-478).
   "We selected to use Ki67 immunolabeling with the goal to determine whether cells are generally proliferative versus quiescent following the various shockwave treatments. Since Ki67 is an endogenous nuclear protein expressed during all active phases of the cell cycle (G1, S, G2, and M), while it is absent in quiescent (G0) cells, this approach was useful for estimating the overall growth fraction of the population of cells."
   Also, we agree, Tuj1 on its own does not support neurogenesis. We have removed those statements from both the abstract and the revised manuscript (old manuscript page: 11, revised manuscript page: 16).

7. **Lack of Additional Functional Markers:** It is surprising that the authors did not analyse cortical actin structures given the morphology observed in Figure 2, nor did they assess neural stress markers such as glutamate transporter or alternative indicators of cell fate and status. I am not sure to understand what they intend to use here as a biomarker.

   Response: We thank the reviewer for this important and insightful comment. We acknowledge that direct visualization of cortical actin structures (e.g., via phalloidin staining) and the inclusion of additional neural stress or fate markers such as glutamate transporters would provide complementary biological validation. However, the primary objective of the present study was to investigate blast-induced cellular responses through nanomechanical and morphological biomarkers obtained using live-cell atomic force microscopy (AFM), rather than molecular or immunocytochemical endpoints. In this work, AFM-derived parameters including changes in cell stiffness (Young's modulus), deformation, surface roughness, and viscoelastic behavior are used as functional, label-free biomarkers that are highly sensitive to cytoskeletal organization. We included the following lines to the revised manuscript with reference to show the correlation of this on page 14 (line: 531-534).
   "Numerous prior studies (Gavara and Chadwick (2016) [43], Codan et al.(2014) [44], and Masud et al. (2025) [60]) have established a strong correlation between actin cytoskeleton remodeling and AFM-measured mechanical properties, where alterations in actin filament density, alignment, and crosslinking directly reflect alterations in cellular stiffness and surface microtopography."
   47. Gavara N, Chadwick RS. Relationship between cell stiffness and stress fiber amount, assessed by simultaneous atomic force microscopy and live-cell fluorescence imaging. Biomechanics and modeling in mechanobiology. 2016 Jun;15(3):511-23.
   48. Codan B, Del Favero GI, Martinelli V, Long CS, Mestroni L, Sbaizero O. Exploring the elasticity and adhesion behavior of cardiac fibroblasts by atomic force microscopy indentation. Materials Science and Engineering: C. 2014 Jul 1;40:427-34.
   32. Masud N, Hasib MH, Ibironke B, Block C, Hughes J, Ekpenyong A, Sarkar A. Exploring the heterogeneity in glioblastoma cellular mechanics using in-vitro assays and atomic force microscopy. Scientific Reports. 2025 Jun 2;15(1):19302.
   The following additional line can be found in the discussion section (page: 14, line: 539-541).
   "Thus, the AFM images and cell surface data presented here provide indirect yet functionally relevant evidence of actin cytoskeletal reorganization following blast exposure."
   Regarding neural stress markers and cell fate indicators, we agree that such analyses would be valuable in extending the biological interpretation of injury mechanisms. However, the scope of the current study was intentionally focused on early-stage, mechanically driven cellular responses immediately following blast exposure, where biophysical alterations often precede detectable changes in molecular expression. To clarify the use of biomarkers in the manuscript, we clarified this as follows on page 17 (line: 651-653).
   "The biomarkers used here therefore represent early, mechanosensitive indicators of cellular stress and injury rather than downstream biochemical signaling events."
