# Supplementary material for: The effects of blast-induced traumatic brain injury on brain cellular mechanics and differentiation
Source: PLoS One. 2026 Aug 13;21(8):e0355739. doi: 10.1371/journal.pone.0355739 (PMC13472447; doi:10.1371/journal.pone.0355739)
Supplement: Supplementary file 11 [file pone.0355739.s012.pdf]

Response to Reviewers

The effects of blast-induced traumatic brain injury on brain cellular mechanics and differentiation

We extend our appreciation to the editor for giving us the opportunity to revise our manuscript, taking into consideration the constructive feedback provided by the reviewer. We hope that with these changes (highlighted in the paper with red color), the paper can be accepted without any further revisions. We have addressed the individual concerns of the reviewers in detail below. We look forward to the comments on our revised version.

Reviewer #1:

We thank the reviewer for the feedback on our manuscript.

The authors have carefully addressed the comments from both reviewers, and the manuscript has been substantially improved. The revisions, including the clarification of the methods and the addition of figures, have enhanced the overall clarity of the study. However, one remaining point relates to the limitations of the experimental model. Reviewer 1 raised concerns regarding the possibility that the shockwave exposure in this experimental system may involve transmission of mechanical energy from air to the liquid culture medium, which may influence how the stimulus is delivered to the cells.

In the current revision, this issue does not appear to be fully addressed. While additional experiments may be beyond the scope of the present study, the authors should clearly acknowledge this limitation and discuss how the air-to-liquid transmission of shockwaves may affect the interpretation of the results. Explicitly stating this limitation would help ensure that the validity and scope of the findings are appropriately framed.

Response: We thank the reviewer for this important suggestion. We agree that the shockwave experienced by the cells was likely modified after propagation through air and transmission into the culture medium. Due to the substantial acoustic impedance mismatch between air and the aqueous culture medium, the incident shockwave undergoes significant reflection and phase inversion at the air-liquid interface. These interactions can generate additional tensile stresses and promote cavitation within the medium, both of which may contribute to the observed cellular responses. In response to the reviewer's concern, we have explicitly acknowledged this limitation by incorporating the following statement into the revised manuscript (page-16, line 621-635):

"We also emphasize that our findings should be interpreted with consideration of the complex shockwave dynamics occurring at the air–liquid interface. Because the acoustic impedance of water (or culture medium) is roughly 3500$\times$ of air [61], the liquid-air interface behaves similarly to a rigid boundary during shockwave transmission. Prior studies showed that when a shock wave strikes an air–water interface, it is predominantly reflected with phase inversion [62]. It results in the conversion of the initial compressive pulse into a tensile (negative-pressure) wave within the liquid phase. Such tensile stresses can substantially increase the likelihood of cavitation. In practice, we expect the incident blast pulse to be strongly attenuated and inverted upon entering the medium. Importantly, we did not measure the shock waveform inside the liquid, so the exact transmitted pressure history at the cells is unknown. Future investigations incorporating in situ pressure measurements and computational modeling of shockwave transmission through the air-liquid interface will be essential for accurately characterizing the mechanical environment at the cellular level."

References:

61 Rajasekar, J., Kim, T. H., & Kim, H. D. (2025). Study of Cylindrical Explosive Shock Waves at Air-Water Interface. Open Journal of Fluid Dynamics, 15(1), 1-18.

62 Dietz-Laursonn, K., Beckmann, R., Ginter, S., Radermacher, K., & de la Fuente, M. (2016). In-vitro cell treatment with focused shockwaves—influence of the experimental setup on the sound field and biological reaction. Journal of therapeutic ultrasound, 4(1), 10.

Reviewer #2:

I would first like to thank the authors for including the PI control, which I believe strengthens the presented results. After reading the rebuttal letter, I now understand that the source of my confusion was the time points used in their experiments.

Figure S1 shows the experimental timeline; however, it is not clear why results are not shown for each time point. In their rebuttal letter, the authors clarify that the final time points correspond to 12 or 10 DIV of total culture time, which is 6–7 DIV post-shockwave exposure. This makes some figure legends ambiguous. For example, the legend of Figure 6 — "Differentiation and Proliferation of AHPCs at 4 DIV Post-Expansion Following Single or Double Shockwave Exposure from "Bottom-to-Top" — could be interpreted as 4 DIV of total culture time, which would imply DIV 0 post-shockwave exposure. The same ambiguity applies to Figure 5, which refers to "6–7 DIV post-expansion." This distinction is critical. Transcription and translation of the markers used here require a minimum of approximately 16 hours from the time of external stimulation to detectable protein levels, and this estimate is conservative for antibody-based detection, which relies on the accumulation of substantial protein quantities. If too little time has elapsed since shockwave exposure, the observed cellular responses may reflect prior manipulations rather than a true response to the shockwave itself. Given that the controls show slightly different results, I suspect this is primarily a phrasing issue rather than an experimental one, but clarification is essential.

I strongly recommend that the authors: (1) consistently label figures and experiments according to both total culture time and time post-shockwave exposure, as depicted in Figure S1, and (2) provide representative images for all time points for which they show in Figure S1, they have PI and ICC coverslips or indicate more clearly which figures contain these images.

Response: We thank the reviewer for highlighting the ambiguity in the expanded experimental timeline and the inconsistent reporting of culture duration across the manuscript. We agree that the phrases we used may have led to confusion in interpreting the experimental design. Taking the Reviewer's suggestions into account, we have revised the manuscript to ensure consistent labeling throughout the Results section, figure captions, and supplementary captions for clarity.

The changes can be found at:

- Page 5, Line 157 (Materials and methods section)
- Page 11, Lines 399, 413, 420 (Results section)
- Page 12, Line 452 (Results section)
- Page 12 (Figure 5 captions)
- Page 13 (Figure 6 captions)
- Page 15, Line 585 (Discussion section)
- Page 17, Lines 678–692 (S2 Figure captions)
- Page 17–18, Lines 693–705 (S3 Figure captions)
- Page 18, Lines 706–728 (S4 Figure captions)
- Page 18, Lines 729–741 (S5 Figure captions)
- Page 18–19, Lines 742–754 (S6 Figure captions)
- Page 19, Lines 755–772 (S7 Figure captions)

We have also updated the Kaggle datasets to match the revised labeling used in the revised manuscript. We believe these revisions resolve the ambiguity and improve consistency and reproducibility throughout the study.
