# Supplementary material for: The effects of blast-induced traumatic brain injury on brain cellular mechanics and differentiation
Source: PLoS One. 2026 Aug 13;21(8):e0355739. doi: 10.1371/journal.pone.0355739 (PMC13472447; doi:10.1371/journal.pone.0355739)
Supplement: Supplementary file 12 [file pone.0355739.s013.pdf]

Response to Reviewers

The effects of blast-induced traumatic brain injury on brain cellular mechanics and differentiation

We extend our appreciation to the editor for giving us the opportunity to revise our manuscript, taking into consideration the constructive feedback provided by the reviewer. We hope that with these changes (highlighted in the paper with red color), the paper can be accepted without any further revisions. We have addressed the individual concerns of the reviewers in detail below. We look forward to the comments on our revised version.

Reviewer #1:

We thank the reviewer for the feedback on our manuscript.

The authors have appropriately revised the manuscript through the initial submission and two rounds of revision. Overall, the wording has become more cautious and scientifically balanced, and the authors appear to have responded sincerely to the reviewers' concerns.

In particular, the revised discussion of the limitations related to shockwave transmission across the air–liquid interface is helpful and improves the interpretation of the experimental model. One additional point that may strengthen the Discussion is the relationship between repeated shockwave exposure and reduced cellular stability.

The present findings that repeated shockwave exposure causes greater impairment of cellular mechanical stability may be discussed in the broader context of repeated traumatic brain injury. In clinical neurotrauma, the concept of repeated concussion and second-impact syndrome illustrates that repeated mechanical insults can produce disproportionately severe biological consequences.

Response: We thank the reviewer for this helpful suggestion. We agree that expanding the Discussion to place our findings within a broader mechanobiological context and to emphasize that repeated shockwave exposure may reduce cellular mechanical stability through a cumulative injury mechanism, rather than a simple linear dose-dependent effect, strengthens the manuscript. Accordingly, we have added the following text to the Discussion (page 15, lines 597–614), as suggested by the reviewer:

"The relatively more severe cellular mechanical instability in response to repetitive shockwave exposure in our model system fits into the general theory of cumulative mechanical vulnerability associated with traumatic brain injury. At the sub-cellular level, a single non-failing sub-threshold mechanical stress can momentarily impair the plasma membrane and cortical cytoskeleton but fails to lead to overt damage due to the ability of the cell to repair itself [62]. The second stress applied before the completion of the repair process causes much greater cytoskeletal impairment compared to one application of stress of similar intensity, as shown in an axon stretch-injury model in which repetitive very mild stretching enhanced growth cone collapse and cytoskeletal mislocalization beyond what was seen after one mild stretch [63]. Similar cumulative damage has been noted in shear-sensitive blood vessels exposed to repetitive blast. This intracellular fragility reflects the clinically recognized condition known as repeat concussion and second impact syndrome, where a second trauma to the head occurring prior to the recovery from the first injury results in an exaggerated biological reaction [64,65]. It appears that there is a similar limited capacity for restoration from sub-failure damage that contributes to decreased cellular resistance to mechanical strain following repeat shock wave application in our study. "

References:

62 Hemphill MA, Dauth S, Yu CJ, Dabiri BE, Parker KK. Traumatic brain injury and the neuronal microenvironment: a potential role for neuropathological mechanotransduction. Neuron. 2015 Mar 18;85(6):1177-92.

63 Yap YC, King AE, Guijt RM, Jiang T, Blizzard CA, Breadmore MC, Dickson TC. Mild and repetitive very mild axonal stretch injury triggers cystoskeletal mislocalization and growth cone collapse. PloS one. 2017 May 4;12(5):e0176997.

64 Laurer HL, Bareyre FM, Lee VM, Trojanowski JQ, Longhi L, Hoover R, Saatman KE, Raghupathi R, Hoshino S, Grady MS, McIntosh TK. Mild head injury increasing the brain's vulnerability to a second concussive impact. Journal of neurosurgery. 2001 Nov 1;95(5):859-70.

65 DeFord SM, Wilson MS, Rice AC, Clausen T, Rice LK, Barabnova A, Bullock R, Hamm RJ. Repeated mild brain injuries result in cognitive impairment in B6C3F1 mice. Journal of neurotrauma. 2002 Apr;19(4):427-38.

Although the present study is an in vitro cellular model and should not be directly equated with clinical concussion, a brief reference to this concept may help readers understand the potential relevance of repeated mechanical loading. Another potentially interesting neurosurgical perspective concerns the directionality of mechanical injury.

The authors showed different cellular responses between top-to-bottom and bottom-to-top shockwave exposure. In vivo, the transmission of mechanical force is likely influenced by cranial anatomy, including differences in skull thickness and skull base structure. For example, in mammals, the skull base is structurally robust and may reflect evolutionary adaptation to protect the brain against forces transmitted from below or through the cranial base.

If relevant anatomical or comparative studies are available, a short discussion of how skull geometry, bone thickness, and direction of impact may modulate intracranial force transmission could further enrich the interpretation of the direction-dependent findings.

However, this should be presented cautiously, as the present experimental system does not directly model the intact skull.

Overall, I consider that the manuscript has been appropriately revised and that the authors have addressed the reviewers' comments in a scientifically responsible manner.

Response: We thank the reviewer for this constructive suggestion. We acknowledge that our in vitro system should not be equated directly with clinical concussion, but that a brief mechanobiological framing is appropriate for interpreting the direction-dependent effects we observed. Taking the Reviewer's suggestions into account, we have therefore added the following in the Discussion section (page:14, line:514-514 & page:14, line:541-545):

"These direction-related cell responses can be understood on the basis of existing biomechanical research, which shows that the shape of the skull is able to control both the direction and focus of mechanical force transmission. Biomechanically, the skull base is mechanically stronger than the skull vault, needing significantly more force and energy to break it, and loading occurs primarily axially rather than radially (for example, via the cervical spine and foramen magnum)[47]. Also, it was demonstrated experimentally that regional bone thickness variations within the skull actively influence the directions of intracranial force vectors, thus resulting in the specific neuropathological patterns that depend on the mechanical load direction [48]. Clinical data show that the direction of force application relative to skull anatomy alone affects the degree of brain damage [49].

While our in vitro system does not include a cranium and cannot be used as an analogy to these force transmission mechanisms, such an anatomical background suggests that *in vivo* similar variations in force direction related to our 'top-to-bottom' and 'bottom-to-top' exposures can be additionally influenced and even amplified/buffered by the mechanical properties of the skull."

47 McELHANEY JH, Hopper JR RH, Nightingale RW, Myers BS. Mechanisms of basilar skull fracture. Journal of neurotrauma. 1995 Aug;12(4):669-78.

48 Beitchman JA, Lifshitz J, Harris NG, Thomas TC, Lafrenaye AD, Hånell A, Dixon CE, Povlishock JT, Rowe RK. Spatial distribution of neuropathology and neuroinflammation elucidate the biomechanics of fluid percussion injury. Neurotrauma Reports. 2021 Feb 8;2(1):neur-2020.

49 Mori M, Aramaki Y, Fukushima K, Oshima K. Evaluation of the Direction of External Force Input to the Skull and Its Influence on the Severity of Traumatic Brain Injury. Cureus. 2025 Oct 30;17(10).

Reviewer #2:

Thank you for revising the experimental nomenclature. The updated labeling makes it much clearer how long the cells were cultured following the shockwave treatment and how each image corresponds to the respective conditions. Overall, the experiments are now significantly easier to interpret.

I noticed a last item that is actually not trivial regarding the statistics used throughout the manuscript, clarification here is also necessary, apologies for not noticing this earlier. The statistical approach is generally appropriate (t-tests/ANOVA for continuous and percentage outcomes), but there are important issues that need clarification and possibly re-analysis. In the ICC and PI assays, the authors report 20–30 image fields per condition and N = 2–3 independent experiments, but it is not clear whether the statistical unit is the image field or the independent culture. If fields from the same coverslip/experiment are treated as independent replicates, this constitutes pseudoreplication; the appropriate n should be the number of independent experiments, with fields or cells treated as technical subsampling. Similarly, for the AFM data the authors report 20 cells and 40 data sets per condition but do not specify how many independent culture preparations contributed these measurements or how the hierarchical structure (cells within dish) was handled. The rationale for using paired t-tests is also unclear, as the manuscript does not describe a clear pairing structure (e.g., same dish measured before and after shock). I recommend that the authors (i) clearly state the level

of replication and the statistical unit for each assay, (ii) justify the use of paired tests in the AFM analyses or switch to appropriate unpaired tests, and (iii) if necessary, re-analyze the data using biological replicate as n (e.g., averaging fields/cells within each experiment) to avoid pseudoreplication and more accurately reflect uncertainty.

Response: We thank the reviewer for this careful and constructive comment. We agree that the statistical unit of analysis and the rationale for paired testing needed to be stated explicitly, and we have revised the manuscript accordingly.

i. We acknowledge the reviewer's concern that the replication structure described in the figure legends was unclear to the reader. To clarify the statistical unit: the percentage of positive staining (Ki67, TuJ1, MAP2ab, RIP, PI) provided for each figure panel (Main manuscript Figs. 5M &N, 6M &N, and supplementary figures) is the average of the percentage of staining per image field, which is calculated as an average of all experiments to give one representative measurement per experimental condition. Then, it was used as input for an unpaired t-test (Welch's correction) or one-way ANOVA with Tukey's multiple comparison test. These 10 image fields per experiment were simply a subsampling for obtaining a reliable estimate of the percentage of positive staining per experiment and were not used as independent replicates for inferential statistical analysis. For this analysis, for the control condition, we only had data from 2 independent replicates from a total of 20 image fields (10 for each replicate). For the shock condition, we had data from 3 independent experiments from a total of 30 image fields (10 for each replicate). Indeed, we would like to note that while this was implied and not explicitly mentioned in the original submission, it caused confusion. Therefore, we have made modifications in the Materials and Methods Section "Data acquisition and statistical analysis" (Page:8, Line:282-307) as follows, as well as all applicable figure legends (S2, S3, S4).

ii. For the AFM nanomechanical measurements (Fig. 3), data for the control condition were obtained from N = 2 independent experiments, whereas data for the single and double shockwave-exposed conditions were obtained from N = 3 independent experiments. It contributes to a total of 20 individually indented cells per condition. The corresponding viscoelastic analysis pooled 40 force-time data points per condition across the same 3 preparations. We would like to clarify that these independent experiments were not technical repeats performed on the same day from a shared cell stock: cells were seeded and prepared independently in three separate weeks, each producing its own control and shockwave-exposed dishes. These are, therefore, independent biological preparations, each contributing a similar number of cells (6–7) to the pooled distribution per condition to make the apparent sample size of 20, not a case of one dominant session inflating an apparent sample size.

We would like to clarify the rationale for using the individual cell, rather than the independent culture, as the statistical unit for the AFM nanomechanical analyses. In ICC/PI, each image field reports a population-averaged readout (percentage of positive cells in that field), so the field is a technical subsample of one biological entity. However, for AFM nanoindentation each cell is indented and resolved individually, and the dominant source of variance in single-cell measurements is well documented in the AFM literature to be cell-to-cell mechanical heterogeneity within a population. For this reason, the 'single-cell AFM mechanics' field typically defines around N = 15 cells as the number of individual cells probed and pooled across the independent preparations that contributed them, rather than as the number of independent cultures. We have revised the Materials and Methods on page 8 (line: 282-307) and the Fig. 3 legend to state this explicitly to avoid the confusion.

"For each independent experiment, ten image fields were acquired for each stain or antibody. Consequently, 20 or 30 image fields per condition were quantified from N = 2 or N = 3 independent experiments, respectively. Then, the percentage of immunoreactive cells was averaged across all these image fields, resulting in one representative measurement per experimental condition. On the other hand, a total of 20 individually indented cells per condition were quantified for the AFM analysis. The control group comprised cells from N = 2 independent experiments, while the shockwave-exposed group comprised cells from N = 3 independent experiments. All means are reported with standard deviation (mean ± SD) or standard error of the mean (mean ± SEM). Graph Pad Prism 10 (Graph Pad Software, Inc., San Diego, CA) was used for ICC/PI, and OriginPro 2022 (64-bit) SR1 version 9.9.0.225 (Academic) was used for AFM statistical analysis and graph-making. Statistical comparisons were conducted on the mean of these replicates (N = 2 or N = 3 replicates per condition), using either an unpaired T-test with Welch's correction (two-group comparisons) or ordinary one-way ANOVA using Tukey's multiple comparisons test, with $\alpha = 0.05$ for both ICC/PI and AFM. The significance levels are denoted as follows: * for $P < 0.05$; ** for $P < 0.01$; *** for $P < 0.001$; and **** for $P < 0.0001$.

The units used for statistical analysis in this study vary depending on the assays and their designs. For example, in the case of the population-averaged parameters (ICC, PI), the unit of analysis is an image field from an independently prepared coverslip, while cells within an image field act as technical sub-samples. In contrast, for 'single-cell AFM nanoindentation' experiments, cells are analyzed individually because intercellular mechanical heterogeneity is a well-known factor in this type of measurement [34,35,36]. In compliance with the standard approach in single-cell AFM

experiments [37,38,39], we therefore used the individual cell, pooled across N = 3 independent preparations, as the statistical unit for the AFM comparisons.

34 Sokolov I, Dokukin ME, Guz NV. Method for quantitative measurements of the elastic modulus of biological cells in AFM indentation experiments. Methods. 2013 Apr 1;60(2):202-13.

35 Guz N, Dokukin M, Kalaparthi V, Sokolov I. If cell mechanics can be described by elastic modulus: study of different models and probes used in indentation experiments. Biophysical journal. 2014 Aug 5;107(3):564-75.

36 Lekka M. Discrimination between normal and cancerous cells using AFM. Bionanoscience. 2016 Mar;6(1):65-80.

37 Cross SE, Jin YS, Rao J, Gimzewski JK. Nanomechanical analysis of cells from cancer patients. InNano-enabled medical applications 2020 Nov 23 (pp. 547-566). Jenny Stanford Publishing.

38 Schillers H, Rianna C, Schäpe J, Luque T, Doschke H, Wälte M, Uriarte JJ, Campillo N, Michanetzis GP, Bobrowska J, Dumitru A. Standardized nanomechanical atomic force microscopy procedure (SNAP) for measuring soft and biological samples. Scientific reports. 2017 Jul 11;7(1):5117.

39 Rother J, Nöding H, Mey I, Janshoff A. Atomic force microscopy-based microrheology reveals significant differences in the viscoelastic response between malign and benign cell lines. Open biology. 2014 May 21;4(5):140046.

We also acknowledge that the original manuscript referred to these comparisons as “paired” T-tests and no individual cell was measured twice (before and after shockwave exposure). The updated analysis compares two pooled, unmatched distributions of cells across conditions. In response to the reviewer’s suggestion, we reran this analysis with unpaired, two-tailed t-tests (Welch’s correction for unequal variances), which is the statistically appropriate test for comparing two independently sampled cell populations. The significance pattern reported in the revised analysis is almost negligible. We updated the individual graphs in Fig. 3 with the updated significance results.

iii. For the reasons already explained in (i) and (ii) responses, since the cell was the standard unit of experiment in single-cell measurements, and field images were the standard unit of experiment in ICC/PI data, we didn’t re-analyze the data using independent cultures. However, we reanalyzed the statistical analysis for the AFM nanoscale structural, mechanical, and viscoelastic data with an unpaired T-test. We can understand the reviewer’s concern regarding 'pseudoreplication', we would like to clarify that this is not valid in our case, as the ICC/ PI/AFM data were collected from separate cultures. We have explicitly clarified in the Materials and Methods section in the revised manuscript, updated the main figure 3 and supplementary figure caption (S2,S3,S4).
