# Supplementary material for: The effects of blast-induced traumatic brain injury on brain cellular mechanics and differentiation
Source: PLoS One. 2026 Aug 13;21(8):e0355739. doi: 10.1371/journal.pone.0355739 (PMC13472447; doi:10.1371/journal.pone.0355739)
Supplement: Peer review history

# Author comment: Author response to Decision Letter 0

**DOI:** 10.1371/journal.pone.0355739.r001

**Transfer Alert**

This paper was transferred from another journal. As a result, its full editorial history (including decision letters, peer reviews and author responses) may not be present.

31 Jul 2025

# Peer review: Decision Letter 0

**DOI:** 10.1371/journal.pone.0355739.r002

**Authors:** Divakar Sharma

25 Nov 2025

Dear Dr. Sarkar,

Thank you for submitting your manuscript to PLOS ONE. After careful consideration, we feel that it has merit but does not fully meet PLOS ONE’s publication criteria as it currently stands. Therefore, we invite you to submit a revised version of the manuscript that addresses the points raised during the review process.

==============================

**ACADEMIC EDITOR: Major Revision Requested**

==============================

Please submit your revised manuscript by Jan 09 2026 11:59PM. If you will need more time than this to complete your revisions, please reply to this message or contact the journal office at plosone@plos.org . When you're ready to submit your revision, log on to https://www.editorialmanager.com/pone/ and select the 'Submissions Needing Revision' folder to locate your manuscript file.

- A rebuttal letter that responds to each point raised by the academic editor and reviewer(s). You should upload this letter as a separate file labeled 'Response to Reviewers'.
- A marked-up copy of your manuscript that highlights changes made to the original version. You should upload this as a separate file labeled 'Revised Manuscript with Track Changes'.
- An unmarked version of your revised paper without tracked changes. You should upload this as a separate file labeled 'Manuscript'.

If you would like to make changes to your financial disclosure, please include your updated statement in your cover letter. Guidelines for resubmitting your figure files are available below the reviewer comments at the end of this letter.

If applicable, we recommend that you deposit your laboratory protocols in protocols.io to enhance the reproducibility of your results. Protocols.io assigns your protocol its own identifier (DOI) so that it can be cited independently in the future. For instructions see: https://journals.plos.org/plosone/s/submission-guidelines#loc-laboratory-protocols . Additionally, PLOS ONE offers an option for publishing peer-reviewed Lab Protocol articles, which describe protocols hosted on protocols.io. Read more information on sharing protocols at https://plos.org/protocols?utm_medium=editorial-email&utm_source=authorletters&utm_campaign=protocols .

We look forward to receiving your revised manuscript.

Kind regards,

Divakar Sharma

Academic Editor

PLOS ONE

Journal Requirements:

When submitting your revision, we need you to address these additional requirements.

1. Please ensure that your manuscript meets PLOS ONE's style requirements, including those for file naming. The PLOS ONE style templates can be found at

https://journals.plos.org/plosone/s/file?id=wjVg/PLOSOne_formatting_sample_main_body.pdf and

https://journals.plos.org/plosone/s/file?id=ba62/PLOSOne_formatting_sample_title_authors_affiliations.pdf

2. Thank you for stating in your Funding Statement:

“This work was supported by Iowa State University Harpole-Pentair Professorship (AS), and Stem Cell Research Support Fund (DSS)”

Please provide an amended statement that declares *all* the funding or sources of support (whether external or internal to your organization) received during this study, as detailed online in our guide for authors at http://journals.plos.org/plosone/s/submit-now. Please also include the statement “There was no additional external funding received for this study.” in your updated Funding Statement.

Please include your amended Funding Statement within your cover letter. We will change the online submission form on your behalf.

3. Thank you for stating the following financial disclosure:

“This work was supported by Iowa State University Harpole-Pentair Professorship (AS), and Stem Cell Research Support Fund (DSS)”

Please state what role the funders took in the study. If the funders had no role, please state: "The funders had no role in study design, data collection and analysis, decision to publish, or preparation of the manuscript."

If this statement is not correct you must amend it as needed.

Please include this amended Role of Funder statement in your cover letter; we will change the online submission form on your behalf.

4. We note that your Data Availability Statement is currently as follows: All materials and data are included within the manuscript and its Supporting Information files and will be available publicly.

Please confirm at this time whether or not your submission contains all raw data required to replicate the results of your study. Authors must share the “minimal data set” for their submission. PLOS defines the minimal data set to consist of the data required to replicate all study findings reported in the article, as well as related metadata and methods (https://journals.plos.org/plosone/s/data-availability#loc-minimal-data-set-definition).

For example, authors should submit the following data:

- The values behind the means, standard deviations and other measures reported;

- The values used to build graphs;

- The points extracted from images for analysis.

Authors do not need to submit their entire data set if only a portion of the data was used in the reported study.

If your submission does not contain these data, please either upload them as Supporting Information files or deposit them to a stable, public repository and provide us with the relevant URLs, DOIs, or accession numbers. For a list of recommended repositories, please see https://journals.plos.org/plosone/s/recommended-repositories.

If there are ethical or legal restrictions on sharing a de-identified data set, please explain them in detail (e.g., data contain potentially sensitive information, data are owned by a third-party organization, etc.) and who has imposed them (e.g., an ethics committee). Please also provide contact information for a data access committee, ethics committee, or other institutional body to which data requests may be sent. If data are owned by a third party, please indicate how others may request data access.

5. We note that you have included the phrase “data not shown” in your manuscript. Unfortunately, this does not meet our data sharing requirements. PLOS does not permit references to inaccessible data. We require that authors provide all relevant data within the paper, Supporting Information files, or in an acceptable, public repository. Please add a citation to support this phrase or upload the data that corresponds with these findings to a stable repository (such as Figshare or Dryad) and provide and URLs, DOIs, or accession numbers that may be used to access these data. Or, if the data are not a core part of the research being presented in your study, we ask that you remove the phrase that refers to these data.

6. We note that Figure 1 in your submission contain copyrighted images. All PLOS content is published under the Creative Commons Attribution License (CC BY 4.0), which means that the manuscript, images, and Supporting Information files will be freely available online, and any third party is permitted to access, download, copy, distribute, and use these materials in any way, even commercially, with proper attribution. For more information, see our copyright guidelines: http://journals.plos.org/plosone/s/licenses-and-copyright.

We require you to either (1) present written permission from the copyright holder to publish these figures specifically under the CC BY 4.0 license, or (2) remove the figures from your submission:

a. You may seek permission from the original copyright holder of Figure 1 to publish the content specifically under the CC BY 4.0 license.

We recommend that you contact the original copyright holder with the Content Permission Form (http://journals.plos.org/plosone/s/file?id=7c09/content-permission-form.pdf) and the following text:

“I request permission for the open-access journal PLOS ONE to publish XXX under the Creative Commons Attribution License (CCAL) CC BY 4.0 (http://creativecommons.org/licenses/by/4.0/). Please be aware that this license allows unrestricted use and distribution, even commercially, by third parties. Please reply and provide explicit written permission to publish XXX under a CC BY license and complete the attached form.”

Please upload the completed Content Permission Form or other proof of granted permissions as an "Other" file with your submission.

In the figure caption of the copyrighted figure, please include the following text: “Reprinted from [ref] under a CC BY license, with permission from [name of publisher], original copyright [original copyright year].”

b. If you are unable to obtain permission from the original copyright holder to publish these figures under the CC BY 4.0 license or if the copyright holder’s requirements are incompatible with the CC BY 4.0 license, please either i) remove the figure or ii) supply a replacement figure that complies with the CC BY 4.0 license. Please check copyright information on all replacement figures and update the figure caption with source information. If applicable, please specify in the figure caption text when a figure is similar but not identical to the original image and is therefore for illustrative purposes only.

7. If the reviewer comments include a recommendation to cite specific previously published works, please review and evaluate these publications to determine whether they are relevant and should be cited. There is no requirement to cite these works unless the editor has indicated otherwise.

Additional Editor Comments:

Major Revision Requested

[Note: HTML markup is below. Please do not edit.]

Reviewers' comments:

Reviewer's Responses to Questions

**Comments to the Author**

1. Is the manuscript technically sound, and do the data support the conclusions?

Reviewer #1: Yes

Reviewer #2: Partly

**********

2. Has the statistical analysis been performed appropriately and rigorously?

Reviewer #1: Yes

Reviewer #2: Yes

**********

3. Have the authors made all data underlying the findings in their manuscript fully available?

The PLOS Data policy

Reviewer #1: Yes

Reviewer #2: No

**********

4. Is the manuscript presented in an intelligible fashion and written in standard English?

Reviewer #1: Yes

Reviewer #2: Yes

**********

Reviewer #1: This manuscript addresses an important topic in blast-induced traumatic brain injury using an in vitro model of adult hippocampal progenitor cells. The study is interesting and the findings are potentially valuable. However, several major points need to be addressed before the manuscript can be considered for publication:

Methods: For basic experimental studies, detailed methodology is essential. The manuscript should provide a clear and comprehensive description of the experimental procedures.

Blast model orientation: The rationale and significance of the “top-to-bottom” versus “bottom-to-top” exposure need to be explicitly stated and discussed in more detail.

Ethical considerations: If animal-derived cells or models were used, the ethical approval and considerations should be described.

Clinical implications: The statements on regenerative medicine and personalized medicine are currently speculative. These should either be removed or supported with appropriate evidence and references.

Figures: A schematic or actual photograph of the blast model setup would greatly help readers understand the experimental system.

Overall, the manuscript has potential, but substantial revision is required to improve scientific rigor, clarity, and presentation.

Reviewer #2: The manuscript by Masud and collaborators investigates the effects of blast-induced traumatic brain injury (bTBI) on adult rat hippocampal progenitor cells using an in vitro model. The researchers employ a custom-built 'Schedule 80' compression-driven shock tube to simulate blast trauma by delivering single or double shockwaves at various pressures and directions. Subsequently, they assess mechanical alterations in cell stiffness and viscosity through atomic force microscopy and evaluate cellular morphology. Immunohistochemical analyses are used to probe changes in cellular function, aiming to reveal cellular resilience and propose new diagnostic or therapeutic avenues for bTBI.

One of the manuscript's notable strengths is the sophisticated and deliberate design of the shock tube system, which facilitates precise simulation of bTBI conditions in vitro. Particularly compelling is the systematic comparison of shockwave directionality (top-to-bottom vs. bottom-to-top), which reveals that acoustic impedance mismatches at the medium interface dictate significant variations in energy transmission and mechanical disruption. The bottom-to-top exposure is designed to minimize energy dissipation, ensuring a more direct impact compared to the top-to-bottom approach. The dose- and orientation-dependency of injury is convincingly corroborated by atomic force microscopy images.

Despite these technical strengths, the characterization of functional cellular outcomes is insufficient and hampered by several methodological weaknesses:

1. Viability Measurement Limitations:The authors' reliance on propidium iodide (PI) staining fails to account for cells lost to detachment following shockwave exposure. Consequently, reported viability rates (>98-99%) reflect only those cells that remain adherent, not the total population initially present. This undermines all viability statistics, given that cell loss is visible as clumping and empty spaces in the panels.

2. Unquantified Immediate Cell Loss: The omission of detached, dead cells from viability counts results in a systematic underestimation of initial trauma-induced damage.

3. In Vitro Model Context: Although controlling for variables, the exclusive use of an in vitro model (with AHPCs) diverges significantly from in vivo conditions. Furthermore, the lack of physical integration of substrates and blast setup introduces the possibility of air blast or sound artifacts affecting cells—calling into question the ecological validity of the experimental system.

4. Limited Long-Term Differentiation Data: While early markers of differentiation are explored at 4 or 6–7 days in vitro (DIV), there is little examination of later-stage differentiation or lineage outcome, limiting conclusions about persistent or functionally relevant changes.

5. Inappropriate Use of DAPI:The manuscript’s use of DAPI as a live-cell indicator is highly unconventional, as live cells do not incorporate DAPI. Complete DAPI positivity indicates cell death, further confounding data interpretation in Figures S2 and S3. Moreover, the lack of clear PI staining is suspicious. Employing a proper death control and established viability assays (e.g., PI and Hoechst 33342 or Calcein/PI combination) would yield more reliable results.

6. Inconclusive Marker Data: Most bar graphs depicting up- or downregulation of markers fail to show robust or interpretable effects; double-labeling approaches may be necessary for clarity. For example, the purported decline in viability following double versus single shockwave exposure (Figure S4) is not substantiated by the data, as all groups display nearly 100% viability. Edu as a measure of proliferation would be much more interpretable than the use of Ki67. Tuj1 on its own does not support neurogenesis. Control experiments are missing here.

7. Lack of Additional Functional Markers: It is surprising that the authors did not analyse cortical actin structures given the morphology observed in Figure 2, nor did they assess neural stress markers such as glutamate transporter or alternative indicators of cell fate and status. I am not sure to understand what they intend to use here as a biomarker.

Collectively, while the manuscript excels in technical execution of shockwave delivery and initial mechanical assessment, its conclusions regarding cell viability, neurogenesis, and functionality are undermined by inadequate methodology and incomplete quantification of cellular responses. A more rigorous and holistic approach to viability, death, and differentiation analysis, alongside the inclusion of functionally relevant markers, would greatly strengthen the manuscript's claims.

**********

what does this mean? ). If published, this will include your full peer review and any attached files.

If you choose “no”, your identity will remain anonymous but your review may still be made public.

**Do you want your identity to be public for this peer review?**   For information about this choice, including consent withdrawal, please see our Privacy Policy

Reviewer #1: No

Reviewer #2: No

**********

[NOTE: If reviewer comments were submitted as an attachment file, they will be attached to this email and accessible via the submission site. Please log into your account, locate the manuscript record, and check for the action link "View Attachments". If this link does not appear, there are no attachment files.]

To ensure your figures meet our technical requirements, please review our figure guidelines: https://journals.plos.org/plosone/s/figures

You may also use PLOS’s free figure tool, NAAS, to help you prepare publication quality figures: https://journals.plos.org/plosone/s/figures#loc-tools-for-figure-preparation.

NAAS will assess whether your figures meet our technical requirements by comparing each figure against our figure specifications.

# Author comment: Author response to Decision Letter 1

**DOI:** 10.1371/journal.pone.0355739.r003

19 Feb 2026

We have uploaded the “Response to Reviewers” as a separate document, as it contains figures and images that cannot be displayed within the system’s text response field. Please refer to the attached “Response to Reviewers” file for the complete and detailed rebuttal.

Attachment Submitted filename: Response to Reviewers.pdf

# Peer review: Decision Letter 1

**DOI:** 10.1371/journal.pone.0355739.r004

**Authors:** Divakar Sharma

28 Apr 2026

Dear Dr. Sarkar,

Thank you for submitting your manuscript to PLOS ONE. After careful consideration, we feel that it has merit but does not fully meet PLOS ONE’s publication criteria as it currently stands. Therefore, we invite you to submit a revised version of the manuscript that addresses the points raised during the review process.

==============================

**ACADEMIC EDITOR:**

==============================

Please submit your revised manuscript by Jun 12 2026 11:59PM. If you will need more time than this to complete your revisions, please reply to this message or contact the journal office at plosone@plos.org . When you're ready to submit your revision, log on to https://www.editorialmanager.com/pone/ and select the 'Submissions Needing Revision' folder to locate your manuscript file.

- A letter that responds to each point raised by the academic editor and reviewer(s). You should upload this letter as a separate file labeled 'Response to Reviewers'.
- A marked-up copy of your manuscript that highlights changes made to the original version. You should upload this as a separate file labeled 'Revised Manuscript with Track Changes'.
- An unmarked version of your revised paper without tracked changes. You should upload this as a separate file labeled 'Manuscript'.

If you would like to make changes to your financial disclosure, please include your updated statement in your cover letter. Guidelines for resubmitting your figure files are available below the reviewer comments at the end of this letter.

If applicable, we recommend that you deposit your laboratory protocols in protocols.io to enhance the reproducibility of your results. Protocols.io assigns your protocol its own identifier (DOI) so that it can be cited independently in the future. For instructions see: https://journals.plos.org/plosone/s/submission-guidelines#loc-laboratory-protocols . Additionally, PLOS ONE offers an option for publishing peer-reviewed Lab Protocol articles, which describe protocols hosted on protocols.io. Read more information on sharing protocols at https://plos.org/protocols?utm_medium=editorial-email&utm_source=authorletters&utm_campaign=protocols .

As the corresponding author, your ORCID iD is verified in the submission system and will appear in the published article. PLOS supports the use of ORCID, and we encourage all coauthors to register for an ORCID iD and use it as well. Please encourage your coauthors to verify their ORCID iD within the submission system before final acceptance, as unverified ORCID iDs will not appear in the published article. *Only*   the individual author can complete the verification step; PLOS staff *cannot*   verify ORCID iDs on behalf of authors.

We look forward to receiving your revised manuscript.

Kind regards,

Divakar Sharma

Academic Editor

PLOS One

Journal Requirements:

If the reviewer comments include a recommendation to cite specific previously published works, please review and evaluate these publications to determine whether they are relevant and should be cited. There is no requirement to cite these works unless the editor has indicated otherwise.

Please review your reference list to ensure that it is complete and correct. If you have cited papers that have been retracted, please include the rationale for doing so in the manuscript text, or remove these references and replace them with relevant current references. Any changes to the reference list should be mentioned in the rebuttal letter that accompanies your revised manuscript. If you need to cite a retracted article, indicate the article’s retracted status in the References list and also include a citation and full reference for the retraction notice.

Additional Editor Comments:

minor revision requested

[Note: HTML markup is below. Please do not edit.]

Reviewers' comments:

Reviewer's Responses to Questions

**Comments to the Author**

Reviewer #1: All comments have been addressed

Reviewer #2: (No Response)

**********

2. Is the manuscript technically sound, and do the data support the conclusions?

Reviewer #1: Yes

Reviewer #2: Partly

**********

3. Has the statistical analysis been performed appropriately and rigorously?

Reviewer #1: Yes

Reviewer #2: I Don't Know

**********

4. Have the authors made all data underlying the findings in their manuscript fully available?

The PLOS Data policy

Reviewer #1: Yes

Reviewer #2: Yes

**********

5. Is the manuscript presented in an intelligible fashion and written in standard English?

Reviewer #1: Yes

Reviewer #2: Yes

**********

Reviewer #1: The authors have carefully addressed the comments from both reviewers, and the manuscript has been substantially improved. The revisions, including the clarification of the methods and the addition of figures, have enhanced the overall clarity of the study.

However, one remaining point relates to the limitations of the experimental model. Reviewer #1 raised concerns regarding the possibility that the shockwave exposure in this experimental system may involve transmission of mechanical energy from air to the liquid culture medium, which may influence how the stimulus is delivered to the cells.

In the current revision, this issue does not appear to be fully addressed. While additional experiments may be beyond the scope of the present study, the authors should clearly acknowledge this limitation and discuss how the air-to-liquid transmission of shockwaves may affect the interpretation of the results.

Explicitly stating this limitation would help ensure that the validity and scope of the findings are appropriately framed.

Reviewer #2: I would first like to thank the authors for including the PI control, which I believe strengthens the presented results. After reading the rebuttal letter, I now understand that the source of my confusion was the time points used in their experiments.

Figure S1 shows the experimental timeline; however, it is not clear why results are not shown for each time point. In their rebuttal letter, the authors clarify that the final time points correspond to 12 or 10 DIV of total culture time, which is 6–7 DIV post-shockwave exposure. This makes some figure legends ambiguous. For example, the legend of Figure 6 — "Differentiation and Proliferation of AHPCs at 4 DIV Post-Expansion Following Single or Double Shockwave Exposure from Bottom-to-Top" — could be interpreted as 4 DIV of total culture time, which would imply DIV 0 post-shockwave exposure. The same ambiguity applies to Figure 5, which refers to "6–7 DIV post-expansion."

This distinction is critical. Transcription and translation of the markers used here require a minimum of approximately 16 hours from the time of external stimulation to detectable protein levels, and this estimate is conservative for antibody-based detection, which relies on the accumulation of substantial protein quantities. If too little time has elapsed since shockwave exposure, the observed cellular responses may reflect prior manipulations rather than a true response to the shockwave itself. Given that the controls show slightly different results, I suspect this is primarily a phrasing issue rather than an experimental one, but clarification is essential.

I strongly recommend that the authors: (1) consistently label figures and experiments according to both total culture time and time post-shockwave exposure, as depicted in Figure S1, and (2) provide representative images for all time points for which they show in Figure S1, they have PI and ICC coverslips or indicate more clearly which figures contain these images.

**********

what does this mean? ). If published, this will include your full peer review and any attached files.

If you choose “no”, your identity will remain anonymous but your review may still be made public.

**Do you want your identity to be public for this peer review?**   For information about this choice, including consent withdrawal, please see our Privacy Policy

Reviewer #1: No

Reviewer #2: No

**********

[NOTE: If reviewer comments were submitted as an attachment file, they will be attached to this email and accessible via the submission site. Please log into your account, locate the manuscript record, and check for the action link "View Attachments". If this link does not appear, there are no attachment files.]

To ensure your figures meet our technical requirements, please review our figure guidelines: https://journals.plos.org/plosone/s/figures

You may also use PLOS’s free figure tool, NAAS, to help you prepare publication quality figures: https://journals.plos.org/plosone/s/figures#loc-tools-for-figure-preparation.

NAAS will assess whether your figures meet our technical requirements by comparing each figure against our figure specifications.

# Author comment: Author response to Decision Letter 2

**DOI:** 10.1371/journal.pone.0355739.r005

21 May 2026

We thank the Academic Editor and reviewers for their valuable comments and constructive feedback. We have carefully revised the manuscript in response to all comments raised during the review process. In particular, we clarified the experimental timeline descriptions throughout the manuscript and associated data repository, revised figure labels and captions for consistency, and added a discussion acknowledging the limitations associated with shockwave transmission across the air–liquid interface in the experimental model.

All changes have been addressed in detail in the accompanying "Response to Reviewers" document, and corresponding revisions have been highlighted in the revised manuscript. We believe that these revisions have substantially strengthened the manuscript and hope that the revised version is now suitable for publication in PLOS ONE.

Attachment Submitted filename: Response_to_Reviewers_auresp_2.pdf

# Peer review: Decision Letter 2

**DOI:** 10.1371/journal.pone.0355739.r006

**Authors:** Divakar Sharma

25 Jun 2026

Dear Dr. Sarkar,

Thank you for submitting your manuscript to PLOS ONE. After careful consideration, we feel that it has merit but does not fully meet PLOS ONE’s publication criteria as it currently stands. Therefore, we invite you to submit a revised version of the manuscript that addresses the points raised during the review process.

==============================

**ACADEMIC EDITOR: Minor Revision Requested**

==============================

Please submit your revised manuscript by Aug 09 2026 11:59PM. If you will need more time than this to complete your revisions, please reply to this message or contact the journal office at plosone@plos.org . When you're ready to submit your revision, log on to https://www.editorialmanager.com/pone/ and select the 'Submissions Needing Revision' folder to locate your manuscript file.

- A letter that responds to each point raised by the academic editor and reviewer(s). You should upload this letter as a separate file labeled 'Response to Reviewers'.
- A marked-up copy of your manuscript that highlights changes made to the original version. You should upload this as a separate file labeled 'Revised Manuscript with Track Changes'.
- An unmarked version of your revised paper without tracked changes. You should upload this as a separate file labeled 'Manuscript'.

If you would like to make changes to your financial disclosure, please include your updated statement in your cover letter. Guidelines for resubmitting your figure files are available below the reviewer comments at the end of this letter.

If applicable, we recommend that you deposit your laboratory protocols in protocols.io to enhance the reproducibility of your results. Protocols.io assigns your protocol its own identifier (DOI) so that it can be cited independently in the future. For instructions see: https://journals.plos.org/plosone/s/submission-guidelines#loc-laboratory-protocols . Additionally, PLOS ONE offers an option for publishing peer-reviewed Lab Protocol articles, which describe protocols hosted on protocols.io. Read more information on sharing protocols at https://plos.org/protocols?utm_medium=editorial-email&utm_source=authorletters&utm_campaign=protocols .

As the corresponding author, your ORCID iD is verified in the submission system and will appear in the published article. PLOS supports the use of ORCID, and we encourage all coauthors to register for an ORCID iD and use it as well. Please encourage your coauthors to verify their ORCID iD within the submission system before final acceptance, as unverified ORCID iDs will not appear in the published article. *Only*   the individual author can complete the verification step; PLOS staff *cannot*   verify ORCID iDs on behalf of authors.

We look forward to receiving your revised manuscript.

Kind regards,

Divakar Sharma

Academic Editor

PLOS One

Journal Requirements:

If the reviewer comments include a recommendation to cite specific previously published works, please review and evaluate these publications to determine whether they are relevant and should be cited. There is no requirement to cite these works unless the editor has indicated otherwise.

Please review your reference list to ensure that it is complete and correct. If you have cited papers that have been retracted, please include the rationale for doing so in the manuscript text, or remove these references and replace them with relevant current references. Any changes to the reference list should be mentioned in the rebuttal letter that accompanies your revised manuscript. If you need to cite a retracted article, indicate the article’s retracted status in the References list and also include a citation and full reference for the retraction notice.

Additional Editor Comments:

Minor Revisions Requested

[Note: HTML markup is below. Please do not edit.]

Reviewers' comments:

Reviewer's Responses to Questions

**Comments to the Author**

Reviewer #1: All comments have been addressed

Reviewer #2: All comments have been addressed

**********

2. Is the manuscript technically sound, and do the data support the conclusions?

Reviewer #1: Yes

Reviewer #2: Yes

**********

3. Has the statistical analysis been performed appropriately and rigorously?

Reviewer #1: Yes

Reviewer #2: No

**********

4. Have the authors made all data underlying the findings in their manuscript fully available?

The PLOS Data policy

Reviewer #1: Yes

Reviewer #2: Yes

**********

5. Is the manuscript presented in an intelligible fashion and written in standard English?

Reviewer #1: Yes

Reviewer #2: Yes

**********

Reviewer #1: The authors have appropriately revised the manuscript through the initial submission and two rounds of revision. Overall, the wording has become more cautious and scientifically balanced, and the authors appear to have responded sincerely to the reviewers’ concerns.

In particular, the revised discussion of the limitations related to shockwave transmission across the air–liquid interface is helpful and improves the interpretation of the experimental model. One additional point that may strengthen the Discussion is the relationship between repeated shockwave exposure and reduced cellular stability.

The present findings that repeated shockwave exposure causes greater impairment of cellular mechanical stability may be discussed in the broader context of repeated traumatic brain injury. In clinical neurotrauma, the concept of repeated concussion and second-impact syndrome illustrates that repeated mechanical insults can produce disproportionately severe biological consequences.

Although the present study is an in vitro cellular model and should not be directly equated with clinical concussion, a brief reference to this concept may help readers understand the potential relevance of repeated mechanical loading. Another potentially interesting neurosurgical perspective concerns the directionality of mechanical injury.

The authors showed different cellular responses between top-to-bottom and bottom-to-top shockwave exposure. In vivo, the transmission of mechanical force is likely influenced by cranial anatomy, including differences in skull thickness and skull base structure. For example, in mammals, the skull base is structurally robust and may reflect evolutionary adaptation to protect the brain against forces transmitted from below or through the cranial base.

If relevant anatomical or comparative studies are available, a short discussion of how skull geometry, bone thickness, and direction of impact may modulate intracranial force transmission could further enrich the interpretation of the direction-dependent findings.

However, this should be presented cautiously, as the present experimental system does not directly model the intact skull.

Overall, I consider that the manuscript has been appropriately revised and that the authors have addressed the reviewers’ comments in a scientifically responsible manner.

Reviewer #2: Thank you for revising the experimental nomenclature. The updated labeling makes it much clearer how long the cells were cultured following the shockwave treatment and how each image corresponds to the respective conditions. Overall, the experiments are now significantly easier to interpret.

I noticed a last item that is actually not trivial regarding the statistics used thought the manuscript, clarification here his also necessary, apologies for not noticing this earlier. The statistical approach is generally appropriate (t‑tests/ANOVA for continuous and percentage outcomes), but there are important issues that need clarification and possibly re‑analysis. In the ICC and PI assays, the authors report 20–30 image fields per condition and N = 2–3 independent experiments, but it is not clear whether the statistical unit is the image field or the independent culture. If fields from the same coverslip/experiment are treated as independent replicates, this constitutes pseudoreplication; the appropriate n should be the number of independent experiments, with fields or cells treated as technical subsampling. Similarly, for the AFM data the authors report 20 cells and 40 data sets per condition but do not specify how many independent culture preparations contributed these measurements or how the hierarchical structure (cells within dish) was handled. The rationale for using paired t‑tests is also unclear, as the manuscript does not describe a clear pairing structure (e.g., same dish measured before and after shock). I recommend that the authors (i) clearly state the level of replication and the statistical unit for each assay, (ii) justify the use of paired tests in the AFM analyses or switch to appropriate unpaired tests, and (iii) if necessary, re‑analyze the data using biological replicate as n (e.g., averaging fields/cells within each experiment) to avoid pseudoreplication and more accurately reflect uncertainty.

**********

what does this mean? ). If published, this will include your full peer review and any attached files.

If you choose “no”, your identity will remain anonymous but your review may still be made public.

**Do you want your identity to be public for this peer review?**   For information about this choice, including consent withdrawal, please see our Privacy Policy

Reviewer #1: **Yes:**  Keiichi abe

Reviewer #2: No

**********

[NOTE: If reviewer comments were submitted as an attachment file, they will be attached to this email and accessible via the submission site. Please log into your account, locate the manuscript record, and check for the action link "View Attachments". If this link does not appear, there are no attachment files.]

To ensure your figures meet our technical requirements, please review our figure guidelines: https://journals.plos.org/plosone/s/figures

You may also use PLOS’s free figure tool, NAAS, to help you prepare publication quality figures: https://journals.plos.org/plosone/s/figures#loc-tools-for-figure-preparation.

NAAS will assess whether your figures meet our technical requirements by comparing each figure against our figure specifications.

# Author comment: Author response to Decision Letter 3

**DOI:** 10.1371/journal.pone.0355739.r007

11 Jul 2026

We appreciate the valuable comments and suggestions provided by the Academic Editor and reviewers. A detailed point-by-point response addressing each comment, along with the corresponding revisions made to the manuscript, has been provided in the attached “Response to Reviewers” file.

Attachment Submitted filename: Response_to_Reviewers_auresp_3.pdf

# Peer review: Decision Letter 3

**DOI:** 10.1371/journal.pone.0355739.r008

**Authors:** Divakar Sharma

26 Jul 2026

The effects of blast-induced traumatic brain injury on brain cellular mechanics and differentiation

PONE-D-25-41733R3

Dear Dr. Sarkar,

We’re pleased to inform you that your manuscript has been judged scientifically suitable for publication and will be formally accepted for publication once it meets all outstanding technical requirements.

Within one week, you’ll receive an e-mail detailing the required amendments. When these have been addressed, you’ll receive a formal acceptance letter and your manuscript will be scheduled for publication.

An invoice will be generated when your article is formally accepted. Please note, if your institution has a publishing partnership with PLOS and your article meets the relevant criteria, all or part of your publication costs will be covered. Please make sure your user information is up-to-date by logging into Editorial Manager at Editorial Manager®  and clicking the ‘Update My Information' link at the top of the page. For questions related to billing, please contact billing support .

If your institution or institutions have a press office, please notify them about your upcoming paper to help maximize its impact. If they’ll be preparing press materials, please inform our press team as soon as possible -- no later than 48 hours after receiving the formal acceptance. Your manuscript will remain under strict press embargo until 2 pm Eastern Time on the date of publication. For more information, please contact onepress@plos.org.

Kind regards,

Divakar Sharma

Academic Editor

PLOS One

Additional Editor Comments (optional):

Accept

Reviewers' comments:

Reviewer's Responses to Questions

**Comments to the Author**

Reviewer #1: All comments have been addressed

**********

2. Is the manuscript technically sound, and do the data support the conclusions?

Reviewer #1: Yes

**********

3. Has the statistical analysis been performed appropriately and rigorously?

Reviewer #1: Yes

**********

4. Have the authors made all data underlying the findings in their manuscript fully available?

The PLOS Data policy

Reviewer #1: Yes

**********

5. Is the manuscript presented in an intelligible fashion and written in standard English?

Reviewer #1: Yes

**********

Reviewer #1: The authors have adequately addressed my previous comments and have appropriately revised the Discussion.

I have no further comments and consider the manuscript suitable for publication.

**********

what does this mean? ). If published, this will include your full peer review and any attached files.

If you choose “no”, your identity will remain anonymous but your review may still be made public.

**Do you want your identity to be public for this peer review?**   For information about this choice, including consent withdrawal, please see our Privacy Policy

Reviewer #1: **Yes:**  keiichi abe

**********

# Editor report: Acceptance letter

**DOI:** 10.1371/journal.pone.0355739.r009

**Authors:** Divakar Sharma

PONE-D-25-41733R3

PLOS One

Dear Dr. Sarkar,

I'm pleased to inform you that your manuscript has been deemed suitable for publication in PLOS One. Congratulations! Your manuscript is now being handed over to our production team.

At this stage, our production department will prepare your paper for publication. This includes ensuring the following:

* All references, tables, and figures are properly cited

* All relevant supporting information is included in the manuscript submission,

* There are no issues that prevent the paper from being properly typeset

You will receive further instructions from the production team, including instructions on how to review your proof when it is ready. Please keep in mind that we are working through a large volume of accepted articles, so please give us a few days to review your paper and let you know the next and final steps.

Lastly, if your institution or institutions have a press office, please let them know about your upcoming paper now to help maximize its impact. If they'll be preparing press materials, please inform our press team within the next 48 hours. Your manuscript will remain under strict press embargo until 2 pm Eastern Time on the date of publication. For more information, please contact onepress@plos.org.

You will receive an invoice from PLOS for your publication fee after your manuscript has reached the completed accept phase. If you receive an email requesting payment before acceptance or for any other service, this may be a phishing scheme. Learn how to identify phishing emails and protect your accounts at https://explore.plos.org/phishing.

If we can help with anything else, please email us at customercare@plos.org.

Thank you for submitting your work to PLOS ONE and supporting open access.

Kind regards,

PLOS ONE Editorial Office Staff

on behalf of

Dr. Divakar Sharma

Academic Editor

PLOS One
